# Dengue in Latin America: Systematic Review of Molecular Epidemiological Trends

**DOI:** 10.1371/journal.pntd.0005224

**Published:** 2017-01-09

**Authors:** José Ramos-Castañeda, Flavia Barreto dos Santos, Ruth Martínez-Vega, Josélio Maria Galvão de Araujo, Graham Joint, Elsa Sarti

**Affiliations:** 1 Instituto Nacional de Salud Publica, Centro de Investigaciones sobre Enfermedades Infecciosas, Morelos, Mexico; 2 Laboratório de Imunologia Viral, Instituto Oswaldo Cruz/ Fundação Oswaldo Cruz, Rio de Janeiro, Brazil; 3 Universidad de Santander, Escuela de Medicina, Santander, Colombia; 4 Laboratório de Biologia Molecular de Doenças Infecciosas e do Câncer, Departamento de Microbiologia e Parasitologia; Instituto de Medicina Tropical do Rio Grande do Norte; Universidade Federal do Rio Grande do Norte, Natal, Brazil; 5 Synercom Ltd, Macclesfield, Cheshire, United Kingdom; 6 Sanofi Pasteur, LATAM, Mexico; University of Texas Medical Branch, UNITED STATES

## Abstract

Dengue, the predominant arthropod-borne viral disease affecting humans, is caused by one of four distinct serotypes (DENV-1, -2, -3 or -4). A literature analysis and review was undertaken to describe the molecular epidemiological trends in dengue disease and the knowledge generated in specific molecular topics in Latin America, including the Caribbean islands, from 2000 to 2013 in the context of regional trends in order to identify gaps in molecular epidemiological knowledge and future research needs. Searches of literature published between 1 January 2000 and 30 November 2013 were conducted using specific search strategies for each electronic database that was reviewed. A total of 396 relevant citations were identified, 57 of which fulfilled the inclusion criteria. All four dengue virus serotypes were present and co-circulated in many countries over the review period (with the predominance of individual serotypes varying by country and year). The number of countries in which more than one serotype circulated steadily increased during the period under review. Molecular epidemiology data were found for Argentina, Bolivia, Brazil, the Caribbean region, Colombia, Ecuador, Mexico and Central America, Paraguay, Peru and Venezuela. Distinct lineages with different dynamics were found in each country, with co-existence, extinction and replacement of lineages occurring over the review period. Despite some gaps in the literature limiting the possibility for comparison, our review has described the molecular epidemiological trends of dengue infection. However, several gaps in molecular epidemiological information across Latin America and the Caribbean were identified that provide avenues for future research; in particular, sequence determination of the dengue virus genome is important for more precise phylogenetic classification and correlation with clinical outcome and disease severity.

## Author Summary

The wide distribution of the mosquito vector and the co-circulation of multiple dengue virus serotypes has led to increases in the incidence of dengue in the Americas, where it is a major public health concern. Identifying molecular epidemiological trends may help to identify the reasons for the re-emergence of dengue across Latin America and the Caribbean, and, in turn, enable disease control and management. We conducted this review using well defined methods to search for and identify relevant research according to predetermined inclusion criteria. The objective was to obtain a clearer understanding of changes occurring within dengue serotypes that have resulted in substantial genetic diversity and the emergence of endemic and epidemic strains in different parts of the region. There remain fundamental gaps in our understanding of the epidemiological and evolutionary dynamics of dengue and its relation with disease, and it is not possible to correlate accurately spatial or temporal trends in disease epidemiology, disease severity, or the genetic diversity of DENV. It is important to maintain comprehensive epidemiological surveillance throughout the region (including sequencing of viral strains) to detect new DENV lineages and to understand the regional patterns of DENV dissemination.

## Introduction

Dengue disease, caused by a dengue virus (DENV), is the predominant arthropod-borne viral disease affecting humans. The primary vector is the *Aedes aegypti (A*. *aegypti*) (Linnaeus) mosquito. Dengue is caused by one of four distinct serotypes (DENV-1, -2, -3 or -4) that are members of the *Flaviviridae* family (genus: *Flavivirus*).

The economic burden and the size of the at-risk population confirm the global importance of dengue infections [1]. The direct and indirect costs of dengue are large, with the worldwide costs of medical care, surveillance, vector control and lost productivity estimated to be approximately US$39 billion per year (2010 base) [2]. In the Americas, the economic and societal costs of dengue have been an estimated at between US$1 billion and US$4 billion each year (2010 base) [3]. Dengue is a major public health concern [4–7]; a recent epidemiology systematic review indicated that the incidence of dengue within the Latin America–Caribbean region increased over the period 1995–2010 (pooled incidence was 72.1/100,000 person-years) [8].

The reasons for the spread of dengue in the tropics and subtropics are complex. Population growth, rapid and unplanned urbanisation of tropical regions with poor sanitary conditions, deterioration of the public health infrastructure, decreased access to health care and inadequate vector-control efforts have also contributed to the increase of disease burden [6]. Globalisation of the economy, international travel (recreational, business, and military) and climate change might also explain the disease expansion [6]. The introduction of *Aedes albopictus*, a secondary vector reported for the first time in the continent in 1985, could also play a role in the maintenance of the virus cycle [6]. The relationship between DENV and other co-circulating arboviruses, such as Zika virus (ZIKV) or chikungunya virus (CHIKV), may also influence human infectivity, diagnosis and reported incidence.

Brathwaite *et al*. have segmented the history of dengue in the region into four phases [9].

- Introduction of dengue in the Americas (1600–1946): the first reports of dengue-like epidemics were in Martinique and Guadeloupe (1635), and Panama (1699) [10–13].
- Continental plan for the eradication of the *A*. *aegypti* (1947–1970): marked by the introduction of the Pan American Health Organization (PAHO) *A*. *aegypti* eradication programme to eliminate yellow fever in the region [14].
- *A*.*aegypti* re-infestation (1971–1999): the regional spread of DENV was associated with the cessation of the PAHO eradication program and the migration of *A*. *aegypti* from the Caribbean into northern South America and Venezuela [15–17] and the subsequent re-infestation of Brazil [18].
- Increased dispersion of *A*. *aegypti* and dengue virus circulation (2000–2010). DENV transmission followed the spread of *A*. *aegypti*, probably aided by population increases. Regional dengue incidence is high, with the simultaneous circulation of several serotypes, frequent epidemics, and many countries where it is endemic [11]. The annual number of reported cases and their severity has risen dramatically [6,19,20] with an endemo-epidemic pattern of outbreaks every three to five years [11,21]. Between 2001 and 2007, >4 million cases were notified in the Americas; the most affected sub-region was South America, particularly Brazil, Ecuador, Colombia, Paraguay and Venezuela [11].

DENV RNA is a single-strand positive-sense genome (approximately 10,700 bases), surrounded by a nucleocapsid covered by a lipid envelope. The genome comprises a single open reading frame (ORF), which is co- and post-translationally cleaved into three structural (capsid [C], pre-membrane [prM] and envelope [E]) and seven non-structural (NS1, NS2A, NS2B, NS3, NS4A, NS4B, NS5) proteins. The precursor polyprotein is flanked by two non-translated regions (59 and 39) [22].

DENV genetic diversity can be attributed to the RNA polymerase, which does not have proofreading activity; it is thought to produce approximately one mutation per round of genome replication [23]. DENV ‘genotypes’ can be defined as virus clusters for which associations could be inferred on epidemiological grounds with sequence divergence ≤6% within the chosen genome region [24]. This definition relies on two factors, the genomic region selected and the length of RNA analysed. Differences across the region in the genotyping methods used by the different researches groups appear to be related to nomenclature rather than real differences in methodology.

**Fig 1:**
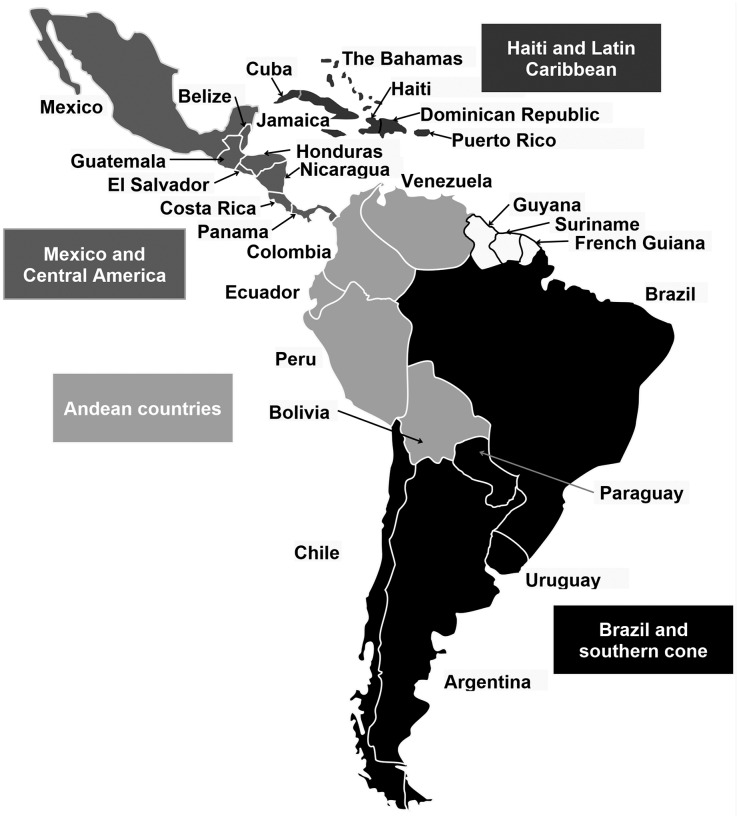
The Latin American region. The strict definition of ‘Latin America’ is the region of the Americas where Latin languages are spoken (Spanish, Portuguese and French). These countries have more in common with each other, sharing elements of historical experience, language and culture, than they do with North America. Nevertheless, Latin America is a diverse group of countries, with varied landscapes, peoples and cultures. However, for the purposes of this review, we included all countries within the Caribbean and Central and South America, and did not exclude countries located in the same region that speak other European languages, such as Belize, Guyana and the smaller Caribbean island nations. Colour code: Black: Brazil and Southern Cone; Dark Grey: Haiti and Latin Caribbean; Mid Grey: Mexico and Central America; Light Grey: Andean countries.

Our systematic review objectives were to describe the molecular epidemiological trends and to identify knowledge gaps relating to dengue disease in Latin America from January 2000 to November 2013.Our definition of ‘Latin America’ included all countries within Central and South America and the Caribbean (Fig 1).

Within some of the phylogenetic studies described in this review, the term ‘lineage’ has been used to describe viruses clustered in clades at a taxonomic level beneath the genotypes [25]. However, as the terms lineage and clade are sometimes used synonymously, we have kept the terminology used in the original sources.

## Materials and Methods

### Search Strategy and Selection Criteria

A literature review group (LRG) guided the systematic review process, defining and preparing the search strategies, protocol and review of documents. Predetermined search strategies were developed, designed to find a high proportion of relevant studies. A number of pilot searches were carried out using terms developed with reference to the expanded Medical Subject Headings (MeSH) thesaurus of the US National Library of Medicine (used for indexing articles in PubMed), after which specific search strategies were devised for each target electronic database.

The following basic search string was employed: dengue AND (Latin America OR Caribbean) AND (molecular epidemiology OR cluster analysis OR dengue/virology OR dengue virus/classification OR dengue virus/genetics OR genotype OR phylogeny OR sequence analysis OR outcomes). To improve accuracy, this was augmented by the addition of specific countries to the generic Latin America and Caribbean terms.

Initial searches were conducted in PubMed, the World Health Organization Library database (WHOLIS); the WHO Regional Database; the Virtual Health Library (VHL; Latin American and Caribbean Center on Health Sciences Information, LILACS); and the Scientific Electronic Library Online (SciELO).

Studies (as well as official reports and bulletins, and conference materials) published in English, Spanish, Portuguese or French between 1 January 2000 and 30 November 2013 were included. References found in databases that did not allow language and/or date limitations and not meeting these criteria were deleted manually at the first review stage. No limits by sex, age and ethnicity of study participants or by study type were imposed, although single-case reports and incomplete surveillance reports (i.e. those with <52 weeks of data) were excluded. As duplicate publications of data (e.g. in meta-analyses and other reviews) could lead to over-sampling, literature reviews and editorials involving previously published peer-reviewed data were also excluded. Publications not identified by the approved search strategy and unpublished data sources meeting the inclusion criteria were included if recommended by LRG members.

Following LRG review of the titles and abstracts, duplicates and articles that did not satisfy the inclusion criteria were removed. Full papers of the first selection of references were retrieved and reviewed as a basis for the final selection. We chose not to exclude articles and other data sources nor formally rank them based on the quality of evidence, as we were of the view that given the nature of molecular epidemiology studies, such quality assessment would not add value to our review.

The selected sources were collated and summarised using a series of Excel (Microsoft Corp., Redmond, WA) spreadsheets. Data from literature reviews of previously published peer-reviewed studies and pre-2000 data published within the search period were not extracted. The original data sources and the extraction tables were made available to all members of the LRG for review and analysis. A meta-analysis was not conducted; a narrative synthesis of our findings is presented.

## Results

**Fig 2:**
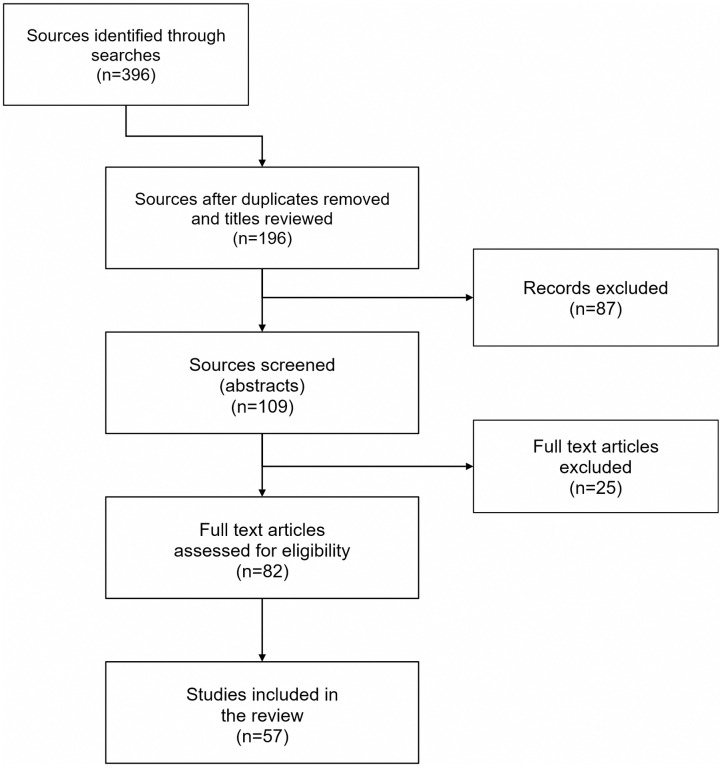
Result of literature search and evaluation of identified studies according to the preferred reporting items of systematic reviews and meta-analyses (PRISMA). All references identified in the on-line database searches were assigned a unique identification number. Following the removal of duplicates and articles that did not satisfy the inclusion criteria from review of the titles and abstracts, the full papers of the first selection of references were retrieved either electronically or in paper form. A further selection was made based on review of the full text of the articles.

The searches identified 396 relevant citations, 57 of which fulfilled the inclusion criteria (Fig 2; and Supplementary S1 Table). The details of the sequencing strategies employed in the studies and other relevant epidemiologic data are included in Supplementary S1 Table.

Data were not found for all countries; published molecular epidemiology data were only identified for Argentina, Bolivia, Brazil, the Caribbean region, Colombia, Ecuador, Mexico and Central America, Paraguay, Peru and Venezuela and these data are reported in this review.

**Fig 3:**
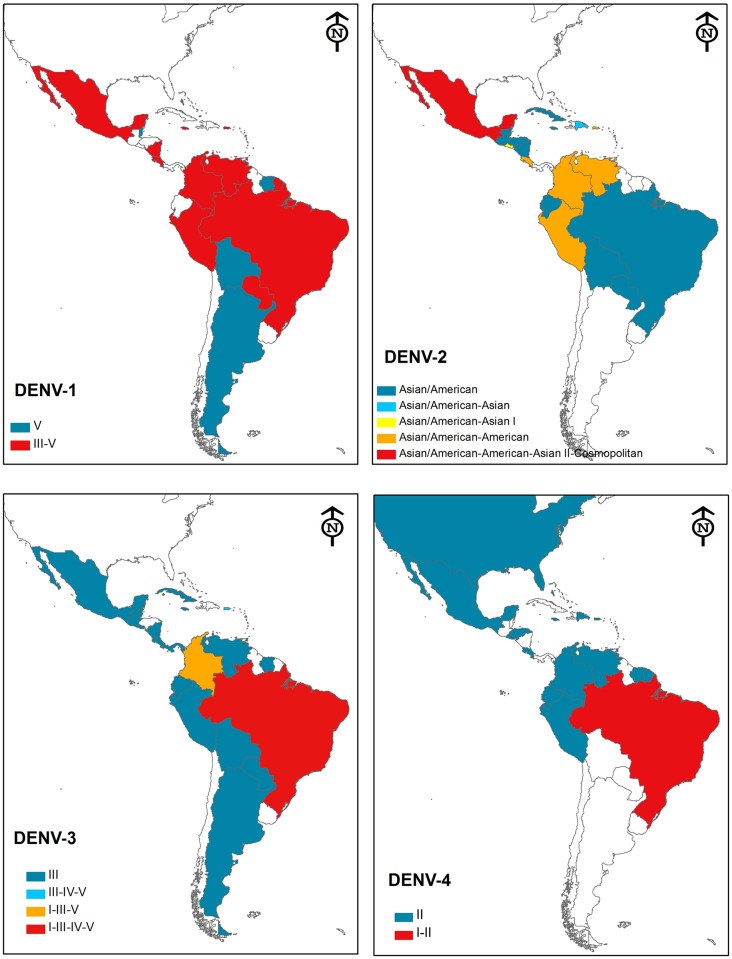
Distribution of DENV genotypes in the Americas region (2002–13). The maps show the reported DENV genotypes by country between 2002 and 2013. The balloons indicate the country where the DENV was isolated, and the colour the genotypes determined. The maps were generated using ArcGIS 10.0 (http://www.strath.ac.uk/is/software/arcgis100-windows/) and modified proportionally to fit in the figure (the bar at the bottom of the panel represents 1,000 km).

Over the review period, the number of countries where more than one DENV serotype was in circulation increased steadily, and all four serotypes were present and co-circulated in many countries [26] (Table 1 [27]). The emergence of the reported genotypes and their geographic distribution are illustrated in the maps (Fig 3). The maps were constructed with the information provided in Supplementary S1 Table, which was elaborated with the information on the phylogenetic trees elaborated in each reference. The majority report the phylogeny based on the E gene sequence although several are based on partial ORF or the complete genome. For DENV-1 and DENV-3, we used the nomenclature for the genotype as it was reported, but we cannot be sure that it represents the same phylogenetic group. This is particularly important in the case of DENV-3 because of the diversity of the genotype in circulation and the fact that the evolution in the Americas region is characterised by replacement; the presence of four genotypes for a relatively newly introduced virus is therefore intriguing.

**Table 1:** Circulating serotypes in the region, according to country for 2000–13 [27].

Year	2000	2001	2002	2003	2004	2005	2006	2007	2008	2009	2010	2011	2012	2013
Region														
**Central America and Mexico**														
Belize	N/A	N/A	2	DEN	3,4	1,2,3	DEN	1	DEN	DEN	DEN	DEN	DEN	DEN
Costa Rica	2	2	1,2	1,2	1,2	1	1,2	1,2	1,2	1,2	1,2,3	1,2,3	1,2,3	1,2,3
El Salvador	2	2	1,2,3,4	2,4	1,2,4	2,4	1,2,4	1,2	DEN	1,2,3,4	1,2	1,2,3,4	1,2,3	1,2,3
Guatemala	2,4	2,4	2,3,4	1,2,3,4	1,2,3,4	1,2,3,4	1,2,4	1,2,4	1,2	2,4	1,2,3,4	1,2	1,2,3,4	1,2,3,4
Honduras	N/A	N/A	2,3,4	2,4	1,2,4	1,2,4	DEN	1,2,4	2,4	1,2	1,2,3,4	1,2	1,2	2,3
Mexico	N/A	N/A	1,2	DEN	1,2,3,4	1,2,3	1	1,2,3,4	1,2,3	1,2,3,4	1,2,3	1,2,3,4	1,2,3,4	1,2,3,4
Nicaragua	2,3	2,3	1,2,4	1	1,2,4	1,2,4	1,2,4	1,2,3	1,2,3,4	1,2,3	1,2,3	1,3	1,2,3	1,2,3,4
Panama	2	2	2	2	1,2,3	1,2	DEN	3	3	1,3	1,3	1,2,3	1,2,3	1,2,3
**Andean**														
Bolivia	1	1	1,2	1,2,3	1,2,3	2,3	2,3	2,3	DEN	1,2,3	1,2	1,2,3	2	1,2,4
Colombia	1,2,4	1,2,4	1,3,4	1,2,3	1,2,3,4	1,2,3	1,2,3,4	1,2,3,4	1,2,3	1,2,3,4	1,2,3,4	1,2,3,4	1,3,4	1,2,3,4
Ecuador	2,3	2,3	2,3	3	1,3,4	1,3	1,3	1,3,4	1,3	1,4	DEN	1,2,4	1,2,4	1,2,4
Peru	1,2,3,4	1,2,3,4	1,3	1,2,3	1,2,3	1,2,3,4	3	1,2,3,4	1,3,4	1,2,3,4	1,2,3,4	1,2,3,4	1,2,3,4	1,2,3,4
Venezuela	1,2,3,4	1,2,3,4	2,3,4	1,2,3	1,2,3,4	1,2,3,4	1,2,3,4	1,2,3,4	1,2,3,4	1,2,3,4	1,2,3,4	1,2,3,4	1,2,3,4	1,2,3,4
**Southern Cone**														
Argentina	AI	AI	1,3	1,2,3	3	2	2,3	2,3	1	1	1,2,4	1,2	1,2,3	1,2,3,4
Brazil	1,2,3	1,2,3	1,2,3	1,2,3	1,2,3	1,2,3	1,2,3	1,2,3	1,2,3	1,2,3	1,2,3,4	1,2,3,4	1,2,3,4	1,2,3,4
Chile	N/A	N/A	N/A	N/A	DEN	DEN	DEN	1	1	1,4	DEN	1	DEN	DEN
Paraguay	1,2	1,2	1,2,3	3	3	2	3	3	DEN	1,3	1,2,3	1,2	2,4	1,2,4
Uruguay	N/A	N/A	N/A	DEN	DEN	DEN	DEN	DEN	DEN	DEN	DEN	DEN	DEN	DEN
**Hispanic Caribbean**														
Cuba	3	3	N/A	DEN	DEN	DEN	DEN	N/A	DEN	DEN	DEN	DEN	DEN	DEN
Dominican Republic	N/A	N/A	2	2	2,4	DEN	1,2	1,2,3,4	DEN	1,2,4	1,2,4	2	2	1,2,4
Puerto Rico	2,3	2,3	2,3	1,2,3	2,3,4	2,3,4	1,2	1,2,3,4	1,2,3	1,3,2,4	1,2,4	1,2,4	1,2,3,4	1,2,4

AI, all imported; DEN, circulating dengue serotypes not specified; N/A, data not available.

Key:

1 circulating serotype: Orange cell; 2 co-circulating serotypes: Blue cell; 3 co-circulating serotypes: Green cell; 4 co-circulating serotypes: Pink Cell.

Source: Pan American Health Organization (2015). Data, Maps and Statistics, Region of the Americas (by country and year). Available: http://www.paho.org/hq/index.php?option=com_topics&view=rdmore&cid=6290&Itemid=40734&lang=en [27].

DENV-1 shows stable circulation of genotype III and circulation of genotype V in the Caribbean region, continent, and islands. Again, it is possible that genotype misclassification can explain the report of genotype V circulation in Argentina and Bolivia. DENV-2 appears to have a high rate of replacement in the Americas region; all the generally accepted genotypes have circulated at some time in the region. However, it should be mentioned that the most virulent genotypes (Asian I and II) are not the most dominant and actually became extinct in certain areas (e.g. Mexico). The South East Asian/American genotype is more dominant than 20 years ago [28]. (Although Asian genotypes have been suggested to produce severe disease, it is intriguing to note that the majority of mortality in the region has been provoked by other DENV types. Of all DENV types, DENV-4 shows high genetic stability and consequently only one genotype [II] has been reported, with the notable exception of the circulation of genotype I in Brazil.)

### Brazil and the Southern Cone

#### DENV-1

DENV-1 was isolated in Brazil between 2009 and 2010, and belonged to genotype V (American/African), grouped in a clade (lineage II) distinct from that of earlier isolates (lineage I); strains isolated in 2011 grouped to form another distinct clade (lineage III) [25]. The introduction of new strains resulted in lineage replacement and an increase in the genetic diversity of DENV-1, but not in positive selection. The dynamics of DENV-1 in Brazil are also characterised by circulation and generation of genetically distinct viruses as a result of local evolution, or exogenous virus introduction during the same period or at different times [29].

DENV-1 isolated in Argentina and Paraguay in 2000 belonged to genotype V (American/African), grouped into two different co-circulating clades and was closely related to viruses from French Guiana (1989), Brazil (1990), and Peru (1997) [30,31]. The virus that circulated in the 1999–2000 outbreak in Paraguay (clade I) was closely related to that present in 1988 [30,31]. In Buenos Aires, only DENV-1 (imported from Paraguay) was detected in 1999–2000, without evidence of local transmission [32].

In Argentina, a ‘wild type + deletion mutant’ mixture was found in two closely related DENV-1 clade II strains isolated in 2000 from the same locality (Eldorado, Misiones Province), characterised by a 3-nucleotide deletion in the NS4A region encoding a 3-amino acid change in the expressed protein [30]. Amino acid differences were also noted between the clades in the C/prM (position 100; Lys→Arg) and E/NS1 (position 722 Ala→Thr) regions [31].

#### DENV-2

Brazilian DENV-2 isolates (n = 25) divided into two epidemiologically distinct groups: 1990–2003 strains (South East Asian genotype, lineage I) with similarities to BR64022/98 (Brazil) and Jamaica/83strains, and 2007–10 strains (South East Asian genotype, lineage II) following the re-emergence of DENV-2 in Brazil. The percentage identity of the latter with strain DR59/01 (Dominican Republic) isolated in 2001, combined with the percentage of divergence with strains first introduced in the 1990s, suggests a new viral lineage introduced from the Caribbean. No consistent differences were observed on the E gene from strains isolated from cases with different clinical manifestations [33].

Another study from Rio de Janeiro (2007–8) showed that those DENV-2 isolates formed a group distinct from the 1990 and 1998 isolates, representing a monophyletic group in the phylogenetic tree with bootstraps of 98%. A temporal circulation of genetically different viruses in Rio de Janeiro was demonstrated that could result from local evolution of DENV-2 or emergence of a new DENV-2 lineage [34]. American/Asian genotype DENV-2 strains isolated during a São Paulo state outbreak (2010) were closely related to strains circulating during the 2007–8 epidemic in Rio de Janeiro and Ribeirão Preto, but distinct from those of earlier DENV-2 epidemics in Brazil. No phylogenetic clustering by clinical presentation was observed [35].

DENV-2 strains (n = 12) from sera of patients with dengue fever (DF) residing in São José do Rio Preto, São Paulo, in 2008 grouped within the American/Asian genotype, together with viruses from South and Central America and the Caribbean. DENV-2 isolates clustered in the BR3 lineage with two Brazilian strains from the northern region and a Jamaican strain isolated in 2007. Similar patterns of introduction/detection were observed for DENV-2 lineages BR1 and BR2 [36].

DENV-2 strains isolated in Paraguay (2001–6) fell into two distinct clades within the American/Asian genotype. Two Paraguayan DENV-2 strains (D2PY-04/01, D2PY-21/05), together with strains from Cuba, the Dominican Republic, Mexico, Nicaragua, and Venezuela that possessed the substitution Glu→Gln at position 131, formed one clade. Another isolate (D2PY-22/05) fell into a separate clade, along with isolates possessing Glu131Leu from Bolivia, Brazil, Colombia, Peru and Venezuela. These findings support the amino acid at position 131 being an important genetic marker for the phylogenetic classification of DENV-2.

#### DENV-3

During the 2002 epidemic in Rio de Janeiro, analysis of 927 acute-phase serum samples showed that 321 of 324 (99.1%) viruses identified were DENV-3 (virus isolation or reverse transcription polymerase chain reaction). Dengue infection was confirmed in 40 (64.5%) of 62 patients who died. Immunohistochemical procedures detected DENV antigen in 48% of specimens from patients with fatal cases, mainly in hepatocytes [37].

DENV-3 isolated from both naturally infected *A*. *aegypti* from Rio de Janeiro and human hosts belonged to genotype III; the majority of isolates were characterised by an 11-nucleotide insertion in the 3′ untranslated region, although strains carrying an 8-nucleotide deletion, or a substitution leading to stop codon formation, were also observed. The role these mutations may play in the transmission of different viral populations and vector competence is unclear [38].

DENV-3 viruses isolated in Porto Velho and Belo Horizonte were closely related to each other and to viruses within genotype V that were isolated in Asia more than 20 years ago [39]. In contrast to official records that DENV-3 was first isolated in Brazil from an autochthonous case in 2000, a DENV-3 E sequence with a high similarity score to the genotype V strains deposited in the GenBank database in 2006 corresponded to a virus isolated in Pará state, Brazil, in 1989. The Brazilian genotype V strains were also unexpectedly similar to the prototype DENV-3 strain identified in the Philippines in 1956 [40]. In another analysis, Brazilian DENV-3 isolates grouped into two separate clades, comprising strains from Acre/Porto Velho/Rio de Janeiro (all genotype III) and Rondônia (genotype V, South East Asia/South Pacific). Co-circulation of genotypes III and V was found in Rondônia [41].

Analysis of DENV-3 viruses from Manaus, Araguaia, Goiania, São Geraldo do São Luis, Cuiabá, Bragança, Iguapé Açu, Marituba, Paranapebas, Santarém, Porto Velho, Boa Vista and Ribeirão Preto isolated in 2002–4 suggested that DENV-3 was introduced into Brazil from the Caribbean at least twice, and into Paraguay from Brazil at least three times [42].

NS5 sequence analysis of isolates from cases during the 2006 São José do Rio Preto outbreak (n = 82) were all DENV-3 and related to strains circulating in Martinique in 2000–1. São José do Rio Preto DENV-3 formed a monophyletic group (lineage 1; n = 60), closely related to the remaining isolates (lineage 2; n = 22). These lineages probably appeared on separate occasions before 2006 [43] and appear to have split one to three years before the last collected sample, propagating in different regions of the city: north-western (lineage 1) and south-eastern (lineage 2) [44]. Although NS5 sequences are more conserved than E sequences (most frequently used in phylogenetic analyses), phylogeny based on NS5 or gene E seems to concur [45].

DENV-3 genotype III has been introduced into Brazil at least four times, although only two lineages (BR-I, BR-II) have become established and disseminated. Three DENV-3 lineages (BR-I, BR-II, BR-III) were probably imported from the Lesser Antilles (Caribbean), while the fourth (BR-IV) was introduced from Colombia or Venezuela. BR-I and -II were most likely introduced via south-eastern and northern Brazil in about 1999 (95% highest posterior density: 1998–2000) and 2001 (95% highest posterior density: 2000–2), respectively, and circulated for one to two years or more until favourable conditions for the initiation of an outbreak occurred [46].

In Argentina, DENV-3 was first characterised in Buenos Aires in 2007 [47]; all isolates belonged to DENV-3 genotype III, those from patients from Brazil (ARG6733-07 and ARG6768-07) clustered apart from those from patients from Paraguay.

In 2001–6, Paraguayan DENV-3 strains were genotype III; several 2006 isolates differed notably from earlier isolates. The most recent DENV-3 isolate, D3PY-27/06, and all pre-2006 Paraguayan strains, clustered into one clade, together with isolates from Brazil and Bolivia. Two other recent DENV-3 strains (D3PY-26/06, D3PY-28/06) and one Brazilian 2004 isolate formed a second clade. D3PY-26/06 and D3PY-28/06 were sampled in Asunción, the most severely affected location during the 2006–7 DENV-3 epidemics. The appearance of new DENV-2 and DENV-3 clades likely produced shifts of dominant serotype in Paraguay in 2005 and 2006), possibly causing the DENV-2 and DENV-3 epidemics in those years [48].

#### DENV-4

Two distinct DENV-4 genotypes (I, II) circulate in Brazil. Analysis confirmed the introduction of DENV-4 genotype I into Brazil from South-East Asia, and at least three introductions of DENV-4 genotype II since 2010: two from Venezuela to Roraima, and one from Colombia to Amazonas. DENV-4 also appears to have been recently introduced into Pará State from the Caribbean region [49].

During the 2011 dengue outbreak, 32% of infections were attributed to DENV-1, with the majority of other infections caused by DENV-4. Phylogenetic analysis revealed the presence of DENV-4 genotype IIb; these strains are closely related to those detected in the city of Roraima in 2010 (strain Br246RR) and São Paulo state in 2011 (strain SPH317947), and strains from Venezuela and Colombia. One characterised strain (RJ1243581) clustered with DENV-4 genotype I and is closely related to strains AM1619 and AM750 from the city of Manaus [50]. In another study, DENV-4 was detected in three samples from patients with suspected dengue infection in Manaus and no travel history, indicating that DENV-4 was autochthonous in Manaus in 2005 [51].

The first autochthonous DENV-4 strains (n = 6) to appear in São Paulo State, Parana State, and Rio Grande do Sul State in 2011 were all genotype II, and closely related to strains circulating in South America since 1981. These monophyletic strains appear to have undergone recent evolution for at least four to six years [52].

### Andean Region

#### DENV-1

Most of the large dengue outbreaks reported in Colombia have involved DENV-1.The majority of isolates analysed belonged to the DENV-1 genotype V (America/Africa), clustered with strains from Brazil, Paraguay, Argentina and the Caribbean; one isolate (probably an introduction that did not spread) was classified as genotype I. A study of the phylogenetic history of DENV-1 has demonstrated two different lineages in Colombia. Throughout the review period, two DENV-1 genotype V lineages have been evolving separately (formed as a result of a split in approximately 1987) [53].

#### DENV-2

DENV-2 American/Asian genotype sequences from Peru are divided into two clearly defined lineages, with lineage II replacing lineage I after 2009. The first Peruvian isolates of the American/Asian DENV-2 genotype were identified during the north-western dengue outbreak in 2000 [54].

Since 2000, the DENV-2 American/Asian genotype in Peru has evolved, with the introduction of four different clades within lineages I and II. Lineages I and II were introduced independently into north-western Peru (via Ecuador, Colombia, and/or Venezuela; lineage I clade A in 2000, lineage II clade E in 2011) and eastern Peru (via Brazil and/or Bolivia; lineage I clade B in 2002, lineage II clade F in 2009). The introduction of new DENV-2 variants belonging to lineage II clade F, a particularly virulent strain, were distinct from those previously circulating in the region, and were possibly associated with a shift in dominant serotypes [55].

American/Asian genotypes circulating from 2002–9 in the Amazon region were more closely related to Brazilian DENV-2 strains, which could be explained by travel between Brazil and Peru facilitating the transmission of these DENV viruses into western Peru [54].

A different lineage of DENV-2 American/Asian genotype, circulating in the city of Iquitos, has been exported to at least four other cities in Peru (Lima, Tarapoto, Trujillo and Yurimaguas). It was reintroduced into Iquitos in late 2010, and was associated with an outbreak of dengue with severe disease and mortality. The Iquitos DENV-2 strains have a very different lineage compared with the circulating virus reported in other countries in Central and South America, with high E/NS1 homology with Brazilian 2007–8 isolates that were also associated with severe cases and deaths. The DENV-2 American/Asian genotype circulating in Peru in 2010 was clearly genetically different from that circulating in 2001, and is associated with severe disease phenotypes [56].

New DENV variants emerged in Bolivia (probably originating from neighbouring countries) during the 2001 DENV-2 and 2007 DENV-1 outbreaks, which were distinct from those identified during previous outbreaks [57].

In Aragua state, Venezuela, only DENV-2 was associated with severe disease, and only one genotype appeared to be circulating for each DENV serotype. However, extensive viral genetic diversity was found in strains isolated from the same area during the same period, indicating significant *in situ* evolution [58].

#### DENV-3

The most recent DENV-3 viruses circulating in South America have evolved to form phylogenetic groups that are distinct from those of Central America. Genetic lineages within DENV-3 subtype III viruses from Venezuela, Peru–Ecuador, and Bolivia–Brazil isolated in 2000–5 may have emerged during or after this serotype’s expansion throughout South America. Four clusters within subtype III were observed: one clade groups the Central American dengue viruses isolated in 1994–98; the second clade comprises Venezuelan isolates from 2001 and 2003; the third group contains isolates from Bolivia, Brazil and Martinique; the fourth node clusters isolates from Ecuador and Peru [59].

DENV-3 genotype I (South-East Asia/South Pacific genotype) was detected co-circulating with genotype III (Indian genotype) in three Colombian states (La Guajira, Guaviare, Huila). Genetic diversity within the 3′ end of the DENV-3 E gene allowed resolution of previous clustering into four lineages (genotypes); the presence of a basal clade in genotype I is consistent with a fifth genotype [60]. Global comparisons of DENV-3 isolates confirmed that Colombian viruses were closely related to those isolated in Asia more than two decades ago [39], and confirmed the presence of genotype V [40]. The identification of genotype V in South American DF/dengue haemorrhagic fever (DHF) samples from 2002–4 is notable because this had been considered an extinct lineage; only three genotype V strains (Philippines/1956/L11423, Japan/1973/ AB11085, and China/1980/AF317645) had been found worldwide before the identification of the South American genotype V strains [40].

Analysis of 2001–7 isolates (n = 21) suggests that Colombian DENV-3 genotype III strains have been introduced from Ecuador, Peru and Venezuela, but not from Argentina, Brazil, Paraguay or Central American countries. Colombian isolates clustered apart from Brazilian isolates, which were associated with a significant number of DHF cases and fatalities [61].

In Ecuador, DENV-3 genotype III strains identified in 2000–5 partitioned into a distinct clade with isolates from Peru. Within this Peru/Ecuador clade, there was no clear segregation between the coastal and jungle isolates from Peru and the isolates from Ecuador, suggesting that a single genotype may be circulating in both countries [59]. Another study found at least six different clades of DENV-3 genotype III circulating across 11 Latin American countries, including Ecuador [62].

A comparison of partial NS5 gene sequences from 23 Ecuadorian DENV strains with 56 DENV strains isolated elsewhere, revealed a close genetic relationship among Ecuadorian strains and Caribbean DENV isolates. Although the Ecuadorian strains did not cluster with the Brazilian DENV-3 isolate (strain EF110568), recent studies suggest that DENV-3 might also have been introduced to Brazil from the Caribbean region [63].

DENV-3 genotype III strains belonging to three different clusters (A to C) were observed in Venezuela, revealing several introduction events. The evolutionary rate for cluster A strains circulating in Venezuela (8.48 x 10^−4^ substitutions/site/year) is similar to that previously established for this genotype in other regions of the world, suggesting a lack of correlation between the DENV-3 genotype III substitution rate and the ecological pattern of virus spread. Amino acid substitution at position 329 of domain III of the E protein (Ala→Val) was found in almost all E proteins from cluster A strains [64].

Determination of the complete sequence of the envelope gene of Venezuelan DENV-3 viruses isolated during 2000 and 2001confirms that the DENV-3 strain currently circulating in the Americas is related to the strain that caused DHF epidemics in Sri Lanka and India in 1989–91 (genotype III). It also appears to be closely related to isolates from Panama and Nicaragua in 1994 that have disseminated throughout Mexico and Central America, and is most closely related to the Mexican isolate from the 1995 epidemic (100% bootstrap support). No evidence was found for re-emergence of the strain that circulated in Venezuela in the late 1960s and 1970s (genotype V) and caused an outbreak [65].

#### DENV-4

Between 2000 and 2006, DENV-4 circulation was rarely detected in Guayaquil, Ecuador and coastal Peru; low-level DENV-4 transmission was detected between 2006 and 2007 in isolates from patients who did not report recent travel outside their respective areas. In February 2008, DENV-4 spread to the Peruvian cities of Iquitos and Yurimaguas, and by October 2008 DENV-4 had nearly completely displaced DENV-3, which had been the only serotype detected in the region during the previous three years.

All DENV-4 isolates were genotype II, although strains from 2006–9 segregated into a markedly different clade from those from 2000. The 2000 isolates clustered more closely with a 1994 Ecuador isolate related to DENV-4 strains introduced from the Caribbean in 1981 (designated as subtype A) [66]. The 2006–9 isolates were most closely related to DENV-4 isolates from Venezuela, with as low as 0.8% nucleotide divergence, and formed a lineage distinct from DENV-4 Caribbean strains, with strong bootstrap support. This lineage is distinguished from previously reported DENV-4 genotype II strains by three conserved amino acid substitutions in the E protein: Ser64Leu, Ala235Thr and Ser403Ala [66].

### Mexico and Central America

#### DENV-1

E gene sequence analysis of Mexican isolates reveals only DENV-1 genotype III, although the presence of three phylogenetically distinct lineages indicates at least three introductions of the genotype, the most recent of which has an inferred date of common ancestry of 1997–2001. These introductions do not appear to be associated with viral lineage co-circulation. The final viral lineage to be introduced circulates in nearly all Mexican states; as isolates are closely related to El Salvador and Nicaragua isolates, they would appear to be dispersed widely around Central America [67].

#### DENV-2

There have been at least 11 separate DENV introductions in Mexico over the past 30 years, with frequent lineage replacement [67]. Lineage co-circulation is relatively limited, and, following lineage replacement, a single viral lineage dominates in a specific serotype at a specific time point [68]. For DENV-2, the emergent Asian/American genotype has established itself as the major lineage in Mexico [67].

At least two DENV-2 American/Asian genotype lineages have circulated in Central America (Belize, Costa Rica, El Salvador, Guatemala, Honduras, Mexico and Nicaragua; 1999–2009). The most recent common ancestor for Central American DENV-2 American/Asian genotype appeared about 1990. There appears to be a region of codons subject to positive selection pressure in the genes encoding for the C, E, NS2A, NS3, and NS5 proteins; some of these codons are novel findings not described previously for any DENV-2 genotype [69].

DENV-2 strains of the American/Asian genotype circulating in Oaxaca, Mexico, are probably from South-East Asia [70]. The 2001 isolates of American/Asian DENV-2 were most similar to the Jamaican and Venezuelan isolates MARA3, LARD1996 and LARD1910. All sequenced strains from Oaxaca possessed a Val residue at position 31 in the prM protein (an amino acid substitution typical of Asian genotype DENV-2). Furthermore, all American/Asian genotypes, and all Oaxaca isolates except HUAT2, have Ile at position 38 in prM, which may be typical for this genotype.

#### DENV-3

In Managua, Nicaragua, sequencing of the ORF in DENV-3 strains from 2008–11 revealed no major genetic changes among yearly isolates [71].

#### DENV-4

Phylogenetic analysis reveals at least two introductions of DENV-4 genotype II viruses into Mexico. Only the second of these occurred during the review period. It comprised a single viral isolate that was newly detected in a 2006 sample [67].

### Caribbean Region

#### DENV-1

Sequencing and phylogenetic analysis of DENV-1 reveal that, as for the other serotypes, DENV-1appears to have arisen from a single introduction prior to the first epidemiological reports of the virus in the region. The population genetic histories of DENV-1 were similar to that of DENV-2 and DENV-4, with an initial invasion phase characterised by rapid increases in genetic diversity, which coincided with the first confirmed cases of each genotype in the region [72]. In Jamaica (2003−07), DENV-1 serotypes were identified in serum samples from suspected dengue. Three samples positive for DENV-1 (3/20, 15%) were identified in 2003 and 2007 [73].

All strains of DENV-1 (n = 3) isolated from patients with dengue in Jamaica during 2007 were classified as genotype III [74]. There has been little evolution in the DENV-1 strains circulating in Jamaica over the years, and this might reduce the risk of outbreaks due to this serotype. In 2010, DENV isolates in Puerto Rico belonged to the American-African genotype (genotype V) to a clade distinct from virus isolated during the 1998 Puerto Rico epidemic. Close ascendants of the 2010 DENV-1 clade had been circulating in Puerto Rico and the Caribbean since at least 2006 [75]. The 2010 DENV-1 strains constitute a new lineage within genotype V different from that circulating in Puerto Rico during the previous two decades [76].

#### DENV-2

Analysis of DENV-2 strains isolated in the Dominican Republic in 2001 showed no amino acid differences between the strains isolated from DF patients (DR23/01 and DR31/01), and DR59/01 isolated from a patient with DHF [77]. All strains had extensive homology with an isolate from Martinique and clustered in the American/Asian genotype. However, Dominican Republic strains and other Caribbean strains from Martinique and Jamaica had 26 amino acid changes that differed from both the Asian and native American genotypes [77].

Comparison of gene sequences of each DENV-2 subtype with those of the DR59/01 (DHF) isolate found that it possessed several codons characteristic of subtype III. Similar amino acid replacements have been observed in the Venezuelan strain Mara 4 isolated from a DHF patient, suggesting the potential for subtype III strains to cause severe forms of DENV infection [77]. In 2010, DENV2 isolates in Puerto Rico (2010) belonged to clade 1B of the American-Asian genotype (genotype IIIb) [75].

#### DENV-3

There is strong evidence for the *in situ* evolution of an Asian DENV-3 genotype III in the Caribbean region since 1994. In Cuba, analysis of the DENV-3 E gene from three patient isolates revealed one genotype III. Comparison of the amino acid sequences of Cuban isolates with other DENV-3 genotype III strains showed that several distinct amino acid replacements had occurred [78]. In Martinique, DENV-3 strains were isolated from patients in 1999–2002 that were closely related to each other and to strains from Sri Lanka (isolated in 2000), the Philippines (reference strain D3PhilH87), Brazil, Guatemala and Mexico. This may suggest a Martinique-specific DENV-3 genotype and a common South-East Asian origin for all DENV-3 strains circulating in the American and Caribbean region [79]. DENV-3 strains isolated during the 2003–4 outbreaks in Saint Martin island (French West Indies) had a common origin with DENV-3 isolated in Martinique two years earlier [80].

Following its re-emergence in Puerto Rico in 1998, the prevalence of an Indian subcontinent strain of DENV-3 increased rapidly over seven years correlating with the disappearance of other serotypes, but its prevalence declined in 2008, and it has not been detected since 2010. Two primary DENV-3 lineages (clades 1 and 2) were identified. Clade 1 consisted of two subclades (1A, 1B), both closely related to international DENV-3 isolates. Clade 2 consisted of multiple subclades, all of which emerged rapidly and almost simultaneously from the parent population. Several subclades (2A, 2E, 2F) also exhibited rapid, sustained diversification [81].

The dissemination pattern of DENV-3 lineages was unlike the evolutionary dynamics of other Puerto Rican serotypes. High mutation rates and rapid replication may have produced a highly heterogeneous population structure at each lineage, but these transient populations appeared to have reduced genetic plasticity and adaptability, ultimately leading to their collapse [81].

#### DENV-4

DENV-4 lineages in the Caribbean and nearby regions appear to be grouped temporally rather than by the geographic origin of isolates. DENV-4 re-emerged in French Guiana, Martinique and Guadeloupe in 2004–5 after a 10-year absence; 2004–5 isolates (n = 5) were genotype II, but of a different clade from strains isolated from French Guiana, Suriname and Costa Rica (1993–95), Martinique (1995) and the Bahamas (1998), suggesting possible evolution from Caribbean island strains from the late 1990s [82]. DENV-4 genotype II has spread rapidly throughout the Caribbean and Latin America and caused DF and sporadic cases of DHF/dengue shock syndrome. It was reintroduced in the French Territories of the Americas in 2004 from neighbouring Caribbean islands [82]. In Puerto Rico, DENV-4 isolates of genotype II are associated with strains that circulated in Puerto Rico throughout the 1980s and 1990s and with strains from the Caribbean region and Central America [76]. Viruses closely-related to the 2010 DENV-4 isolate were first detected in 2004 [75].

## Discussion

Our review provides a comprehensive overview of the evolving molecular epidemiology of dengue disease in Latin America over the period 2000−13. Our literature search was thorough; we screened almost 400 sources to identify relevant data and we developed a comprehensive data extraction instrument to facilitate the capture of those data. However, our review does have some limitations. We could only review published data found by our search techniques and we recognise that some isolated reports from some countries may have escaped our attention and that unusual findings may have a more prominent place in the literature than mainstream findings. Furthermore, our searches may have obscured any bias (by country and time) although we included sources (as well as official reports and bulletins, and conference materials) available in English, Spanish, Portuguese or French over a long time period to reduce the level of selection and publication bias. In addition, we did not formally rank articles and other data sources based on the quality of evidence and any limitations of the original studies are carried forward into our review.

Co-circulation of different serotypes in a region may be a factor in the association between dengue infection and the severity of disease. All four dengue serotypes were found circulating in Latin America, with predominance of individual serotypes varying by country and year. Under these circumstances, populations may exhibit a wide range of humoral and cellular immune responses to DENV, which may increase the likelihood of severe dengue. One such example is the emergence in north-eastern Peru in 2010 of the virulent DENV-2 lineage II clade F, which was associated with the largest DHF epidemic experienced in the region [55]. Although consideration should also be given to the possible effects of co-infections in patients with dengue, these are relatively uncommon. During epidemics, it does happen that some patients have concurrent serotype infections when more than one DENV serotype is being transmitted, but there have been few studies assessing the clinical impact of DENV co-infections. Reports of ZIKV/DENV and CHIKV/DENV co-infections are similarly scarce. Recent studies described a co-infection DENV-2 and ZIKV, isolated from a patient with travel to Haiti who developed fever, rash, arthralgia, and conjunctivitis [83] and a pregnant woman from Colombia with a triple co-infection caused by DENV, CHIKV AND ZIKV [84].

The picture is complicated by multiple introductions of viral lineages of each serotype throughout the countries of Latin America. Intra-serotype antigenic variation and the resulting differential generation of protective antibodies and immune responses is postulated as one of the reasons for the high epidemiological impact of certain DENV serotypes in the Americas [60]. The lack of consistent differences in the E gene of Brazilian DENV-2 isolates from cases with different clinical manifestations suggests that if disease severity has a genetic basis, it is not solely due to differences observed in the E gene [33].

Distinct lineages with different dynamics were identified in each of the countries and co-existence, extinction and replacement of lineages occurred over the review period. Phylogenetic studies suggest that geographical micro-evolution may be operating with regional foci of virus extinction and selection. For example, the Caribbean is the main source of DENV-3 viruses introduced into Brazil, and the northern and south-eastern Brazilian regions seem to be important hubs for the dissemination of those DENV-3 lineages. Phylogenetic analysis also highlights the role played by intensive exchange between bordering countries, particularly that due to tourism and trade [47].

Viral lineages that have circulated in Mexico appear to have the same evolutionary origin as those detected more widely in the Americas (particularly in countries geographically close to Mexico), indicating considerable local diffusion across borders [67]. The resulting pattern of dengue evolution can present an increased risk for subsequent epidemics and severe disease. Further examples are the cultural and economic influence on viral spread and gene flow between the French Territories, the Caribbean and South America [82], and the effect of high viral genetic diversity and a large naive population on the expansion and collapse of DENV-3 in Puerto Rico [81]. Although regions adjacent to the cluster radius might have been protected by herd immunity or cross-protection from other serotypes, re-introduction of a particular DENV serotype or lineage replacement after a period of prolonged absence would expose to infection a population with no immunity to dengue [82].

Micro-evolutionary changes within dengue serotypes have resulted in substantial genetic diversity, with emergence of endemic and epidemic strains. One explanation for the interval between the introduction of DENV in a region and its subsequent detection could be that new DENV genotypes or lineages remain undetected until the number of infections and/or disease incidence reaches a threshold of detection that is high enough to be detected by the local surveillance system. In Brazil, the clustering of DENV-2 isolates in the São José do Rio Preto BR3 lineage (2008) with two strains from the northern region and a 2007 Jamaican strain support suggestions that this lineage was circulating in the country before its first detection in 2007, possibly remaining undetected due to poor surveillance [36]. Similarly, DENV-4 may have re-emerged in Brazil before 2010, but was undetected due to a higher prevalence of DENV-1 and DENV-2, and the failure of the surveillance system to identify the milder disease commonly associated with DENV-4 [52]. These situations highlight the importance of systematic surveillance of dengue viruses because cryptic introduction or circulation of a new DENV serotype into endemic areas is generally considered to increase the risk for severe dengue disease.

Regional extinction and emergence of new lineages is associated with periodic dengue outbreaks and possibly also with the severity and fatality of the disease. The exact cause and effect of viral virulence and lineage changes is yet to be proven, although the extinction of earlier strains and the appearance of new epidemic strains suggest a genetic bottleneck as a cause of regional replacement. Nevertheless, the relative inferiority of the American genotype of DENV-2 in Mexico, coupled with the non-overlapping nature of lineage distributions, suggests that more attention should be paid to the possible role of natural selection in determining patterns of lineage turnover. One hypothesis to explain the possible virulence of emerging clades in the region is an improved ability to avoid neutralisation by serotypes’ cross-reactive antibodies [85].

Although vector competence may be expected to influence the emergence of new lineages, overall many events other than vector competence influence DENV evolution. While vector competence and DENV evolution have been directly tested, no interaction has been demonstrated to date [86], although in some cases enhanced clade transmission was demonstrated in the mosquito [87]. Furthermore, although, genotypes with higher rates of replication are preferentially transmitted by mosquitoes when they are co-infected [88], DENV replication in mosquitoes results in less diversity than in humans [89]. Nevertheless, additional studies are needed to assess whether mosquito vector-driven selection plays a significant role in DENV micro-evolution.

Despite some gaps in the molecular epidemiological information limiting the possibility for comparison, our review has described the molecular epidemiological trends of dengue infection across Latin America and the Caribbean. However, fundamental gaps in our understanding of epidemiological and evolutionary dynamics and its relation with disease remain.

Although the molecular characterisation of DENV strains in a number of the studies included in this review has identified mutations or polymorphisms that appear to be characteristic of specific serotypes, or are associated with increased clinical severity [30,77], it is not possible to correlate accurately spatial or temporal trends in disease epidemiology, disease severity or the genetic diversity of DENV. Sequence determination of the DENV genome is important for more precise phylogenetic classification and correlation with clinical outcome and disease severity. Although it is unlikely that DENV genetic variation will completely explain the incidence of severe disease or the scale of outbreaks, differences in virulence are apparent among DENV lineages [38]. However, how those distinct viral populations are maintained or transmitted is not fully understood.

Additionally, a clearer understanding of the spatial and temporal dynamics of dengue transmission, particularly whether specific lineages are spreading more rapidly than others, will add to our knowledge of local transmission patterns. Furthermore, appreciating the processes that increase the diversity of DENV lineages and the pathogenicity associated with genetic variation will improve our understanding of the mechanisms that govern epidemic development. It may also be of use in vaccine development [30], particularly with regard to the effect of partially effective vaccines on viral population, structure and virulence [90].

Finally, a consistent approach to genotyping would aid cross-study comparisons. One limitation relates to the definition of the DENV ‘genotype’ and its reliance on the genomic region selected and the length of RNA analysed. The genomic region affects sensitivity because it seems that when a partial, usually short, region of the genome (e.g. the E-NS1 or domain III of the E gene) is analysed, the number of genotypes is equal or lower than the result of analysing the entire E gene or the full genome sequence [23]. The length proportionally affects the resolution of tree branches, which could have a high impact if phylodynamic analysis is pursued [91]; for taxonomic purposes, the length also affects sensitivity. In general, as passage through a laboratory culture medium may aid the introduction of mutations [92,93], it is also desirable to use samples that have not undergone laboratory culture to generate amplicon-sequencing data, particularly in viral evolution studies [92], that are not representative of circulating viruses [94]. In addition, the software used should be selected according to the target sequence. For example, the BEAST suite (a cross-platform program for Bayesian analysis of molecular sequences) [95] is currently the best for full genome sequencing or E gene, while E/NS1 junction or partial NS5 can be analysed with the phylogeny model of R software (see https://www.r-project.org).

Overall, the impact of the genomic region selected or the length of the sequence analysed is not critical for a descriptive study; however, when a more detailed study is done (such as phylodynamics or phylogeographic analyses), bigger is better. In this review, we found the heterogeneity introduced from the classification used by a particular group for their findings to be more critical some groups assign numbers to the clusters determined according to their phylogeny, which could prevent comparison with the findings of other groups that may have assigned different numbers. This is an important consideration when trying to make data available for healthcare professionals who do not have a molecular analysis background.

It should also be recognised that the use of sequences in the included studies that were downloaded from GenBank and other public domains may constitute a further limitation in that information about geographical origin or how they were sequenced may be incomplete and thus may compromise any conclusions drawn, which might be based on incomplete or faulty data. It is important that future sequencing methodologies are revised and optimised, with the objective of providing high quality data for future reference.

Finally, if we are to identify spatial or temporal trends in disease epidemiology, disease severity or the genetic diversity of DENV, one target must be the development of a network of national/regional laboratories using the same protocols, reagents and hardware and, importantly, the elaboration of controls, standards and calibrators required for a proper quality control. The Network of PAHO/WHO Collaborating Centers and National Reference Laboratories for Dengue in the Americas (http://www1.paho.org/english/ad/dpc/cd/den-cc.htm) is currently undertaking an initiative to strengthen diagnostic laboratory networks in the region and promote exchange and technological transfer among them.

However, one issue for future surveillance programmes will be to establish the most informative resolution level (e.g. type, genotype, clade) and whether there are any particular mutations that it is important to determine. As there are no odds ratios associated with specific clades or mutations, this would suggest that phylogeography should meet the resolution requirements at the present time.

Another significant obstacle to standardising surveillance methods remains sampling. Although some countries have specific regulations (e.g. Mexican regulations require testing 10% of all dengue cases for virology surveillance and 100% for severe dengue cases), the level of under-sampling is very high. Moreover, there is no information available to make power calculations about the number of samples needed to determinate associations among virus clades and dengue incidence. Consequently, although the PAHO/WHO network will facilitate the monitoring of the introduction and spread of the clades and determine the hot spots of dengue control, it will be extremely difficult, and possibly not economically viable, to build a robust, meaningful molecular virology surveillance system that will answer all the outstanding questions relating to dengue molecular epidemiology.

## Conclusion

In conclusion, the re-emergence of dengue in the Americas may be ascribed jointly to i) the spread of different DENV serotypes in bordering countries, ii) the permanent migration flow of viremic travellers, and iii) an increase in vector infestation due to inconstant vector-control strategies and economic support. In addition, urbanisation has probably had the most impact on the increase of dengue within a country, and the high frequency of low-cost international travel has had the greatest effect on the global spread of dengue [96]. Moreover, DENV seems to take advantage of diverse mechanisms to generate genetic diversity via genetic variability and the (mainly human) movement of the host, as well as exploiting the increasing density of human hosts and urbanisation.

Continuous epidemiological surveillance (employing consistent reporting, definitions and terminology) and sequencing of viral strains circulating in all countries of the region are important for the detection of new DENV lineages and to improve understanding about the regional patterns of DENV dissemination.

## Supporting Information

S1 Table Systematic review evidence table and summary of serological/genotypic data. (PDF)

S1 Checklist PRISMA Checklist. (PDF)

## References

[1] World Health Organization. Global Strategy for Dengue Prevention and Control: 2012–2020. Geneva: WHO; 2012 pp. 1–43.

[2] Selck FW, Adalja AA, Boddie CR. An estimate of the global health care and lost productivity costs of dengue. Vector Borne Zoonotic Dis 2014;14: 824–826. 10.1089/vbz.2013.1528 25409275

[3] Shepard DS, Coudeville L, Halasa YA, Zambrano B, Dayan GH. Economic impact of dengue illness in the Americas. Am J Trop Med Hyg 2011;84: 200–207. 10.4269/ajtmh.2011.10-0503 21292885 PMC3029168

[4] Guzman MG, Kouri G. Dengue and dengue hemorrhagic fever in the Americas: lessons and challenges. J Clin Virol 2003;27: 1–13. 10.1016/s1386-6532(03)00010-6 12727523

[5] Guzman A, Isturiz RE. Update on the global spread of dengue. Int J Antimicrob Agents 2010;36 Suppl 1: S40–S42. 10.1016/j.ijantimicag.2010.06.018 20833000

[6] San Martin JL, Brathwaite O, Zambrano B, Solorzano JO, Bouckenooghe A, Dayan GH, et al The epidemiology of dengue in the americas over the last three decades: a worrisome reality. Am J Trop Med Hyg 2010;82: 128–135. 10.4269/ajtmh.2010.09-0346 20065008 PMC2803522

[7] Isturiz RE, Gubler DJ, Brea del CJ. Dengue and dengue hemorrhagic fever in Latin America and the Caribbean. Infect Dis Clin North Am 2000;14: 121–140, ix 10738676 10.1016/s0891-5520(05)70221-x

[8] Cafferata ML, Bardach A, Rey-Ares L, Alcaraz A, Cormick G, Gibbons L, et al Dengue epidemiology and burden of disease in Latin America and the Caribbean: a systematic review of the literature and meta-analysis. Value Health Reg Issues 2013;2: 347–356. 29702769 10.1016/j.vhri.2013.10.002

[9] Brathwaite DO, San Martin JL, Montoya RH, del DJ, Zambrano B, Dayan GH. The history of dengue outbreaks in the Americas. Am J Trop Med Hyg 2012;87: 584–593. 10.4269/ajtmh.2012.11-0770 23042846 PMC3516305

[10] Gubler DJ. Dengue and dengue hemorrhagic: its history and resurgence as a global public health problem In: Gubler DJ, Kuno G, editors. Dengue and Dengue Hemorrhagic Fever. London: CAB International; 1997 pp. 1–22.

[11] Schneider J, Droll DA. Time Line for Dengue in the Americas to December 31, 2000 and Noted First Occurrences. 2001. www.paho.org/English/HCP/HCT/VBD/dengue_finaltime.doc.

[12] Halstead SB. Dengue: overview and history In: Pasvol G, editor. Tropical Medicine: Science and Practice. London: Imperial College Press; 2008 pp. 1–28.

[13] Badii MH, Landeros J, Cerna E, Abreu JL. Ecología e historia del dengue en las Américas (Ecology and history of dengue in Americas). Daena: International Journal of Good Conscience 2007;2: 248–273.

[14] Pan American Health Organization (PAHO). Directing Council Resolution CD1 R1. PAHO 1947. http://iris.paho.org/xmlui/bitstream/handle/123456789/1733/CD1.R1en.pdf?sequence=1.

[15] Pinheiro FP, Corber SJ. Global situation of dengue and dengue haemorrhagic fever, and its emergence in the Americas. World Health Stat Q 1997;50: 161–169. 9477544

[16] Gubler DJ. Dengue/dengue hemorrhagic fever in the Americas: prospects for the year 2000 In: Halstead SB, Gomez-Dantes H, editors. Dengue, a worldwide problem, a common strategy. Mexico City: Ministry of Health; 1992 pp. 19–27.

[17] Barreto ML, Teixeira MG. Dengue in Brazil: epidemiological situation and contributions for a research agenda. Estud Av 2008;22: 53–72.

[18] Amaral R, Tauil PL. Duas ameaças de um mosquito: febre amarela e dengue. Saúde Bras 1983;4: 236–238.

[19] Pan American Health Organization (PAHO). Number of reported cases of dengue and dengue hemorrhagic fever (DHF), Region of the Americas (by country). PAHO 2013. http://www.paho.org/hq/index.php?option=com_topics&view=readall&cid=3273&Itemid=40734&lang=en.

[20] Teixeira MG, Siqueira JB Jr., Ferreira GL, Bricks L, Joint G. Epidemiological trends of dengue disease in Brazil (2000–2010): a systematic literature search and analysis. PLoS Negl Trop Dis 2013;7: e2520 10.1371/journal.pntd.0002520 24386496 PMC3871634

[21] Guzman Tirado MG, Kouri FG, Bravo Gonzalez JR. [Emergence of dengue hemorrhagic fever in the Americas. Reemergence of dengue]. Rev Cubana Med Trop 1999;51: 5–13. 10887549

[22] Leitmeyer KC, Vaughn DW, Watts DM, Salas R, Villalobos I, de Chacon, et al Dengue virus structural differences that correlate with pathogenesis. J Virol 1999;73: 4738–4747. 10233934 10.1128/jvi.73.6.4738-4747.1999 PMC112516

[23] Chen R, Vasilakis N. Dengue--quo tu et quo vadis? Viruses 2011;3: 1562–1608. 10.3390/v3091562 21994796 PMC3187692

[24] Rico-Hesse R. Molecular evolution and distribution of dengue viruses type 1 and 2 in nature. Virology 1990;174: 479–493. 2129562 10.1016/0042-6822(90)90102-w

[25] dos Santos FB, Nogueira FB, Castro MG, Nunes PC, de Filippis AM, Faria NR, et al First report of multiple lineages of dengue viruses type 1 in Rio de Janeiro, Brazil. Virol J 2011;8: 387 10.1186/1743-422X-8-387 21813012 PMC3170301

[26] Pan American Health Organization (PAHO). Key facts on vector borne diseases dengue. PAHO 2014. http://www.paho.org/hq/index.php?option=com_docman&task=doc_view&gid=24719&Itemid=.

[27] Pan American Health Organization (PAHO). Data, Maps and Statistics, Region of the Americas (by country and year). PAHO 2015. http://www.paho.org/hq/index.php?option=com_topics&view=rdmore&cid=6290&Itemid=40734&lang=en.

[28] Weaver SC, Vasilakis N. Molecular evolution of dengue viruses: contributions of phylogenetics to understanding the history and epidemiology of the preeminent arboviral disease. Infect Genet Evol 2009;9: 523–540. 10.1016/j.meegid.2009.02.003 19460319 PMC3609037

[29] Drumond BP, Mondini A, Schmidt DJ, Bosch I, Nogueira ML. Population dynamics of DENV-1 genotype V in Brazil is characterized by co-circulation and strain/lineage replacement. Arch Virol 2012;157: 2061–2073. 10.1007/s00705-012-1393-9 22777179

[30] Aviles G, Meissner J, Mantovani R, St JS. Complete coding sequences of dengue-1 viruses from Paraguay and Argentina. Virus Res 2003;98: 75–82. 14609632 10.1016/j.virusres.2003.08.018

[31] Aviles G, Rowe J, Meissner J, Manzur Caffarena JC, Enria D, St JS. Phylogenetic relationships of dengue-1 viruses from Argentina and Paraguay. Arch Virol 2002;147: 2075–2087. 10.1007/s00705-002-0886-3 12417945

[32] Barrero PR, Mistchenko AS. Complete genome sequencing of dengue virus type 1 isolated in Buenos Aires, Argentina. Virus Res 2004;101: 135–145. 10.1016/j.virusres.2003.12.033 15041181

[33] Faria NR, Nogueira RM, de Filippis AM, Simoes JB, Nogueira FB, da Rocha Queiroz LM, et al Twenty years of DENV-2 activity in Brazil: molecular characterization and phylogeny of strains isolated from 1990 to 2010. PLoS Negl Trop Dis 2013;7: e2095 10.1371/journal.pntd.0002095 23516646 PMC3597488

[34] Oliveira MF, Galvao Araujo JM, Ferreira OC Jr., Ferreira DF, Lima DB, Santos FB, et al Two lineages of dengue virus type 2, Brazil. Emerg Infect Dis 2010;16: 576–578. 10.3201/eid1603.090996 20202456 PMC3322019

[35] Romano CM, de Matos AM, Araujo ES, Villas-Boas LS, da Silva WC, Oliveira OM, et al Characterization of Dengue virus type 2: new insights on the 2010 Brazilian epidemic. PLoS One 2010;5: e11811 10.1371/journal.pone.0011811 20676363 PMC2911371

[36] Drumond BP, Mondini A, Schmidt DJ, de Morais Bronzoni RV, Bosch I, Nogueira ML. Circulation of different lineages of Dengue virus 2, genotype American/Asian in Brazil: dynamics and molecular and phylogenetic characterization. PLoS One 2013;8: e59422 10.1371/journal.pone.0059422 23533624 PMC3606110

[37] Nogueira RM, Schatzmayr HG, de Filippis AM, dos Santos FB, da Cunha RV, Coelho JO, et al Dengue virus type 3, Brazil, 2002. Emerg Infect Dis 2005;11: 1376–1381. 10.3201/eid1109.041043 16229765 PMC3310608

[38] de Castro MG, de Nogueira FB, Nogueira RM, Lourenco-de-Oliveira R, dos Santos FB. Genetic variation in the 3' untranslated region of dengue virus serotype 3 strains isolated from mosquitoes and humans in Brazil. Virol J 2013;10: 3 10.1186/1743-422X-10-3 23282086 PMC3547765

[39] Aquino VH, Amarilla AA, Alfonso HL, Batista WC, Figueiredo LT. New genotype of dengue type 3 virus circulating in Brazil and Colombia showed a close relationship to old Asian viruses. PLoS One 2009;4: e7299 10.1371/journal.pone.0007299 19823677 PMC2757910

[40] de Araujo JM, Bello G, Schatzmayr HG, Santos FB, Nogueira RM. Dengue virus type 3 in Brazil: a phylogenetic perspective. Mem Inst Oswaldo Cruz 2009;104: 526–529. 19547883 10.1590/s0074-02762009000300021

[41] Nogueira MB, Stella V, Bordignon J, Batista WC, Borba L, Silva LH, et al Evidence for the co-circulation of dengue virus type 3 genotypes III and V in the Northern region of Brazil during the 2002–2004 epidemics. Mem Inst Oswaldo Cruz 2008;103: 483–488. 18797763 10.1590/s0074-02762008000500013

[42] Aquino VH, Anatriello E, Goncalves PF, da Silva EV, Vasconcelos PF, Vieira DS, et al Molecular epidemiology of dengue type 3 virus in Brazil and Paraguay, 2002–2004. Am J Trop Med Hyg 2006;75: 710–715. 17038699

[43] Mondini A, de Moraes Bronzoni RV, Nunes SH, Chiaravalloti NF, Massad E, Alonso WJ, et al Spatio-temporal tracking and phylodynamics of an urban dengue 3 outbreak in Sao Paulo, Brazil. PLoS Negl Trop Dis 2009;3: e448 10.1371/journal.pntd.0000448 19478848 PMC2682200

[44] Nogueira ML, Mondini A, Bronzoni RVM, Alonso WJ, Assad EM, Lazaro E, et al Spatio-temporal and molecular analyses of a DENV3 outbreak show the dynamics of dengue infection (viral spread) [abstract]. Int J Infect Dis 2008;12(Suppl 1): e333.

[45] Schmidt DJ, Pickett BE, Camacho D, Comach G, Xhaja K, Lennon NJ, et al A phylogenetic analysis using full-length viral genomes of South American dengue serotype 3 in consecutive Venezuelan outbreaks reveals a novel NS5 mutation. Infect Genet Evol 2011;11: 2011–2019. 10.1016/j.meegid.2011.09.010 21964598 PMC3565618

[46] de Araujo JM, Bello G, Romero H, Nogueira RM. Origin and evolution of dengue virus type 3 in Brazil. PLoS Negl Trop Dis 2012;6: e1784 10.1371/journal.pntd.0001784 22970331 PMC3435237

[47] Barrero PR, Mistchenko AS. Genetic analysis of dengue virus type 3 isolated in Buenos Aires, Argentina. Virus Res 2008;135: 83–88. 10.1016/j.virusres.2008.02.013 18400327

[48] Aquino JD, Tang WF, Ishii R, Ono T, Eshita Y, Aono H, et al Molecular epidemiology of dengue virus serotypes 2 and 3 in Paraguay during 2001–2006: the association of viral clade introductions with shifting serotype dominance. Virus Res 2008;137: 266–270. 10.1016/j.virusres.2008.07.011 18692099

[49] Nunes MR, Faria NR, Vasconcelos HB, Medeiros DB, Silva de Lima CP, Carvalho VL, et al Phylogeography of dengue virus serotype 4, Brazil, 2010–2011. Emerg Infect Dis 2012;18: 1858–1864. 10.3201/eid1811.120217 23092706 PMC3559147

[50] Campos RM, Veiga CS, Meneses MD, de Souza LM, Fernandes CA, Malirat V, et al Emergence of Dengue virus 4 genotypes II b and I in the city of Rio de Janeiro. J Clin Virol 2013;56: 86–88. 10.1016/j.jcv.2012.10.006 23127562

[51] Figueiredo RM, Naveca FG, Bastos MS, Melo MN, Viana SS, Mourao MP, et al Dengue virus type 4, Manaus, Brazil. Emerg Infect Dis 2008;14: 667–669. 10.3201/eid1404.071185 18394292 PMC2570911

[52] de Souza RP, Rocco IM, Maeda AY, Spenassatto C, Bisordi I, Suzuki A, et al Dengue virus type 4 phylogenetics in Brazil 2011: looking beyond the veil. PLoS Negl Trop Dis 2011;5: e1439 10.1371/journal.pntd.0001439 22216365 PMC3246447

[53] Mendez JA, Usme-Ciro JA, Domingo C, Rey GJ, Sanchez JA, Tenorio A, et al Phylogenetic history demonstrates two different lineages of dengue type 1 virus in Colombia. Virol J 2010;7: 226 10.1186/1743-422X-7-226 20836894 PMC2944171

[54] Cruz CD, Felices V, Aguilar PV, Guevara C, Céspedes M, Suarez V, et al Molecular epidemiology of dengue 2 virus in peru [abstract]. Am J Trop Med Hyg 2010;83 (Suppl 5): 28.

[55] Cruz CD, Forshey BM, Juarez DS, Guevara C, Leguia M, Kochel TJ, et al Molecular epidemiology of American/Asian genotype DENV-2 in Peru. Infect Genet Evol 2013;18: 220–228. 10.1016/j.meegid.2013.04.029 23648427

[56] Mamani E, Alvarez C, Garcia MM, Figueroa D, Gatti M, Guio H, et al [Circulation of a different lineage of dengue virus serotype 2 American / Asian genotype in the Peruvian Amazon, 2010]. Rev Peru Med Exp Salud Publica 2011;28: 72–77. 21537772 10.1590/s1726-46342011000100011

[57] Roca Y, Baronti C, Revollo RJ, Cook S, Loayza R, Ninove L, et al Molecular epidemiological analysis of dengue fever in Bolivia from 1998 to 2008. Vector Borne Zoonotic Dis 2009;9: 337–344. 10.1089/vbz.2008.0187 19505253 PMC3496373

[58] Rodriguez-Roche R, Villegas E, Cook S, Poh Kim PA, Hinojosa Y, Rosario D, et al Population structure of the dengue viruses, Aragua, Venezuela, 2006–2007. Insights into dengue evolution under hyperendemic transmission. Infect Genet Evol 2012;12: 332–344. 10.1016/j.meegid.2011.12.005 22197765 PMC3919160

[59] Kochel T, Aguilar P, Felices V, Comach G, Cruz C, Alava A, et al Molecular epidemiology of dengue virus type 3 in Northern South America: 2000–2005. Infect Genet Evol 2008;8: 682–688. 10.1016/j.meegid.2008.06.008 18674640

[60] Usme-Ciro JA, Mendez JA, Tenorio A, Rey GJ, Domingo C, Gallego-Gomez JC. Simultaneous circulation of genotypes I and III of dengue virus 3 in Colombia. Virol J 2008;5: 101 10.1186/1743-422X-5-101 18764951 PMC2553081

[61] Villabona-Arenas CJ, Miranda-Esquivel DR, Jimenez RE. Phylogeny of dengue virus type 3 circulating in Colombia between 2001 and 2007. Trop Med Int Health 2009;14: 1241–1250. 10.1111/j.1365-3156.2009.02339.x 19619216

[62] de Mora D, Andrea LD, Alvarez M, Regato M, Fajardo A, Recarey R, et al Evidence of diversification of dengue virus type 3 genotype III in the South American region. Arch Virol 2009;154: 699–707. 10.1007/s00705-009-0343-7 19322636

[63] Regato M, Recarey R, Moratorio G, de M D, Garcia-Aguirre L, Gonzalez M, et al Phylogenetic analysis of the NS5 gene of dengue viruses isolated in Ecuador. Virus Res 2008;132: 197–200. 10.1016/j.virusres.2007.10.012 18063164

[64] Ramirez A, Fajardo A, Moros Z, Gerder M, Caraballo G, Camacho D, et al Evolution of dengue virus type 3 genotype III in Venezuela: diversification, rates and population dynamics. Virol J 2010;7: 329 10.1186/1743-422X-7-329 21087501 PMC2998486

[65] Uzcategui NY, Comach G, Camacho D, Salcedo M, Cabello de Q M, Jimenez M, et al Molecular epidemiology of dengue virus type 3 in Venezuela. J Gen Virol 2003;84: 1569–1575. 10.1099/vir.0.18807-0 12771427

[66] Forshey BM, Morrison AC, Cruz C, Rocha C, Vilcarromero S, Guevara C, et al Dengue virus serotype 4, northeastern Peru, 2008. Emerg Infect Dis 2009;15: 1815–1818. 10.3201/eid1511.090663 19891873 PMC2857240

[67] Carrillo-Valenzo E, Danis-Lozano R, Velasco-Hernandez JX, Sanchez-Burgos G, Alpuche C, Lopez I, et al Evolution of dengue virus in Mexico is characterized by frequent lineage replacement. Arch Virol 2010;155: 1401–1412. 10.1007/s00705-010-0721-1 20549264

[68] Diaz FJ, Black WC, Farfan-Ale JA, Lorono-Pino MA, Olson KE, Beaty BJ. Dengue virus circulation and evolution in Mexico: a phylogenetic perspective. Arch Med Res 2006;37: 760–773. 10.1016/j.arcmed.2006.02.004 16824937

[69] Añez G, Morales-Betoulle ME, Rios M. Circulation of different lineages of dengue virus type 2 in Central America, their evolutionary time-scale and selection pressure analysis. PLoS One 2011;6: e27459 10.1371/journal.pone.0027459 22076162 PMC3208639

[70] Cisneros A, Diaz-Badillo A, Cruz-Martinez G, Tovar R, Ramirez-Palacios LR, Jimenez-Rojas F, et al Dengue 2 genotypes in the state of Oaxaca, Mexico. Arch Virol 2006;151: 113–125. 10.1007/s00705-005-0595-9 16096709

[71] Gutierrez G, Standish K, Narvaez F, Perez MA, Saborio S, Elizondo D, et al Unusual dengue virus 3 epidemic in Nicaragua, 2009. PLoS Negl Trop Dis 2011;5: e1394 10.1371/journal.pntd.0001394 22087347 PMC3210753

[72] Allicock OM, Lemey P, Tatem AJ, Pybus OG, Bennett SN, Mueller BA, et al Phylogeography and population dynamics of dengue viruses in the Americas. Mol Biol Evol 2012;29: 1533–1543. 10.1093/molbev/msr320 22319149 PMC3529620

[73] Brown MG, Salas RA, Vickers IE, Heslop OD, Smikle MF. Dengue virus serotypes in Jamaica, 2003–2007. West Indian Med J 2011;60: 114–119. 21942112

[74] Brown M.G., Salas RA, Vickers IE, Heslop OD, Smikle MF. Molecular epidemiology of dengue in Jamaica dengue virus genotypes in Jamaica, 2007. West Indian Med J 2011;60: 120–125. 21942113

[75] Sharp TM, Hunsperger E, Santiago GA, Munoz-Jordan JL, Santiago LM, Rivera A, et al Virus-specific differences in rates of disease during the 2010 Dengue epidemic in Puerto Rico. PLoS Negl Trop Dis 2013;7: e2159 10.1371/journal.pntd.0002159 23593526 PMC3617145

[76] Anez G, Heisey DA, Espina LM, Stramer SL, Rios M. Phylogenetic analysis of dengue virus types 1 and 4 circulating in Puerto Rico and Key West, Florida, during 2010 epidemics. Am J Trop Med Hyg 2012;87: 548–553. 10.4269/ajtmh.2012.12-0091 22826483 PMC3435362

[77] Anzai S, Fukuda M, Otsuka Y, Eshita Y. Nucleotide sequence and phylogenetic analyses of dengue type 2 virus isolated in the Dominican Republic. Virus Genes 2004;29: 219–227. 10.1023/B:VIRU.0000036382.77987.84 15284482

[78] Rodriguez-Roche R, Alvarez M, Holmes EC, Bernardo L, Kouri G, Gould EA, et al Dengue virus type 3, Cuba, 2000–2002. Emerg Infect Dis 2005;11: 773–774. 10.3201/eid1105.040916 15898173 PMC3320353

[79] Peyrefitte CN, Couissinier-Paris P, Mercier-Perennec V, Bessaud M, Martial J, Kenane N, et al Genetic characterization of newly reintroduced dengue virus type 3 in Martinique (French West Indies). J Clin Microbiol 2003;41: 5195–5198. 10.1128/JCM.41.11.5195-5198.2003 14605161 PMC262480

[80] Peyrefitte CN, Pastorino BA, Bessaud M, Gravier P, Tock F, Couissinier-Paris P, et al Dengue type 3 virus, Saint Martin, 2003–2004. Emerg Infect Dis 2005;11: 757–761. 10.3201/eid1105.040959 15890134 PMC3320377

[81] Santiago GA, McElroy-Horne K, Lennon NJ, Santiago LM, Birren BW, Henn MR, et al Reemergence and decline of dengue virus serotype 3 in Puerto Rico. J Infect Dis 2012;206: 893–901. 10.1093/infdis/jis426 22740715 PMC3501150

[82] Dussart P, Lavergne A, Lagathu G, Lacoste V, Martial J, Morvan J, et al Reemergence of dengue virus type 4, French Antilles and French Guiana, 2004–2005. Emerg Infect Dis 2006;12: 1748–1751. 10.3201/eid1211.060339 17283628 PMC3372336

[83] Iovine NM, Lednicky J, Cherabuddi K, Crooke H, White SK, Loeb JC, et al Coinfection With Zika and Dengue-2 Viruses in a Traveler Returning From Haiti, 2016: Clinical Presentation and Genetic Analysis. Clin Infect Dis. 2016 9 29. pii: ciw667. 10.1093/cid/ciw667 PMC6394129 27694479

[84] Villamil-Gómez WE, Rodríguez-Morales AJ, Uribe-García AM, González-Arismendy E, Castellanos JE, Calvo EP, et al Zika, dengue, and chikungunya co-infection in a pregnant woman from Colombia. Int J Infect Dis. 2016 10;51:135–138. 10.1016/j.ijid.2016.07.017 27497951

[85] Kochel TJ, Watts DM, Halstead SB, Hayes CG, Espinoza A, Felices V, et al Effect of dengue-1 antibodies on American dengue-2 viral infection and dengue haemorrhagic fever. Lancet 2002;360: 310–312. 10.1016/S0140-6736(02)09522-3 12147378

[86] Fansiri T, Pongsiri A, Klungthong C, Ponlawat A, Thaisomboonsuk B, Jarman RG, et al No evidence for local adaptation of dengue viruses to mosquito vector populations in Thailand. Evol Appl 2016;9: 608–618. 10.1111/eva.12360 27099625 PMC4831462

[87] Lambrechts L, Fansiri T, Pongsiri A, Thaisomboonsuk B, Klungthong C, Richardson JH, et al Dengue-1 virus clade replacement in Thailand associated with enhanced mosquito transmission. J Virol 2012;86: 1853–61. 10.1128/JVI.06458-11 22130539 PMC3264336

[88] Cologna R, Armstrong PM, Rico-Hesse R. Selection for virulent dengue viruses occurs in humans and mosquitoes. J Virol 2005;79: 853–859. 10.1128/JVI.79.2.853-859.2005 15613313 PMC538581

[89] Vasilakis N, Deardorff ER, Kenney JL, Rossi SL, Hanley KA, Weaver SC. Mosquitoes put the brake on arbovirus evolution: experimental evolution reveals slower mutation accumulation in mosquito than vertebrate cells. PLoS Pathog 2009;5: e1000467 10.1371/journal.ppat.1000467 19503824 PMC2685980

[90] Gandon S, Mackinnon MJ, Nee S, Read AF. Imperfect vaccines and the evolution of pathogen virulence. Nature 2001;414: 751–756. 10.1038/414751a 11742400

[91] Colijin C, Gardy J. Phylogenetic tree shapes resolve disease transmission patterns. Evol Med Public Health 2014;1: 96–108. 10.1093/emph/eou018 PMC4097963 24916411

[92] Chen WJ, Wu HR, Chiou SS. E/NS1 modifications of dengue 2 virus after serial passages in mammalian and/or mosquito cells. Intervirology 2003;46: 289–95. doi: 73208 14555849 10.1159/000073208

[93] Lee E, Weir RC, Dalgarno L. Changes in the dengue virus major envelope protein on passaging and their localization on the three-dimensional structure of the protein. Virology 1997;232: 281–90. 10.1006/viro.1997.8570 9191841

[94] Cruz CD, Torre A, Troncos G, Lambrechts L, Leguia M. Targeted full-genome amplification and sequencing of dengue virus types 1–4 from South America. J Virol Methods 2016;235: 158–67. 10.1016/j.jviromet.2016.06.001 27334982

[95] Drummond AJ, Suchard MA, Xie D, Rambaut A. Bayesian phylogenetics with BEAUti and the BEAST 1.7. Mol Biol Evol 2012;29: 1969–1973. 10.1093/molbev/mss075 22367748 PMC3408070

[96] Wilder-Smith A, Gubler DJ. Geographic expansion of dengue: the impact of international travel. Med Clin North Am 2008;92: 1377–1390. 10.1016/j.mcna.2008.07.002 19061757

