# Supplementary material for: Dengue in Latin America: Systematic Review of Molecular Epidemiological Trends
Source: PLoS Negl Trop Dis. 2017 Jan 9;11(1):e0005224. doi: 10.1371/journal.pntd.0005224 (PMC5221820; doi:10.1371/journal.pntd.0005224)
Supplement: S1 Table — (PDF) [file pntd.0005224.s001.pdf]

Source: first author, year [Ref]	Region/ geographical area	Study type	Study design	Data period: date range/ year	If study:				Summary of data presentation or results/conclusion
					No. patients/ population studied (M:F)	Diagnosis (DF/DHF/ DSS etc.)	Serotype/ genotype (lineage)	Age range	
Allicock 2012 [72]	Antigua*, Aruba, Bahamas, Barbados*, Belize*, Grenada*, Jamaica, St Vincent and the Grenadines*, Suriname*, Trinidad and Tobago*<br>*Sequences derived from 2000 onwards; all were DENV-3	Phylo-geography and population dynamics	Region: E-gene<br>Size sequence: not shown<br>Sequence number to compare: data sets were down-sampled to no more than five sequences per country per year. For DENV-1 V 109, DENV-2 Asian-American 191, DENV-3 III 226, and DENV-4 II 214<br>Software: BEAST v1.6.1 software package	1977–2005	DENV-1 (n=18) and DENV-3 (n=25) strains isolated from patient sera and DENV-1 genotype 5, DENV-2 American/ Asian genotype, DENV-3 genotype III, and DENV-4 genotype II sequences from GenBank		DENV-1 genotype V<br>DENV-2 Asian–American genotype<br>DENV-3 genotype III<br>DENV-4 genotype II		All four DENV serotypes appear to have arisen from a single introduction prior to the first epidemiological reports of the virus in the region. The population genetic histories of DENV-1, DENV-2, and DENV-4 were similar, with an increase in genetic diversity upon introduction, followed by a maintenance phase during which genetic diversity remained stable with only gradual increases or decreases depending on serotype. DENV-3 genotype III had a different population genetic history, with no obvious maintenance phase
Aquino 2006 [42]	Brazil (Manaus–AM, Araguaia–GO, Goiania–GO, Saõ Geraldo do Saõ Luis–MA, Cuiabá–MT, Bragança–PA, Iguapé Açu-PA, Marituba–PA, Paranapebas–PA, Santarém–PA, Porto Velho–RO, Boa Vista–RR, Ribeiraõ Preto–SP), Paraguay (Asunción, Fernando de la Mora, Pedro Juan	Molecular epidemio-logical	Region: the E protein gene and the 3′ and 5′ UTRs<br>Size sequence: 1,479 nucleotides of E gen., 94 of 5′ UTRs<br>Sequence number to compare: 45 for E gen., 22 for 3′ UTRs<br>Software: PAUP 4.0b8a software (Sinauer, Sunderland, MA)	2002–2004	DENV-3 strains from patients with DF (n=27) or fatal DHF/DSS (n=1)	DF	DENV-3, genotype III		DENV-3 was introduced into Brazil from the Caribbean islands at least twice, and into Paraguay from Brazil at least three times. DENV-3 circulating in Brazil and Paraguay grouped with other American viruses and viruses isolated in Sri Lanka and Samoa, belonging to genotype III

Source: first author, year [Ref]	Region/ geographical area	Study type	Study design	Data period: date range/ year	If study:				Summary of data presentation or results/conclusion
					No. patients/ population studied (M:F)	Diagnosis (DF/DHF/ DSS etc.)	Serotype/ genotype (lineage)	Age range	
	Caballero, Yaguarón)								
Aquino 2008 [48]	Paraguay (Asunción, Ciudad del Este, Fernando de la Mora, Hernandarias, Itauguá, Luque, Pedro J. Caballero, Yaguarón)	Molecular epidemio-logical	Open: Bayesian phylogenetic analysis of the nucleotide sequencing of the complete E-gene of 4 x DENV-2 and 22 x DENV-3 strains. Sequencing method: the RT-PCR purified products were sequenced with the BigDye® Terminator v1.1 Cycle Sequencing Kit (Applied Biosystems), followed by purification using	2001–2006	DENV-2 (n=4) and DENV-3 (n=22) strains isolated from patient sera		DENV-2 American/Asian genotype (2 distinct clades possessing either Q or L at E131) DENV-3 genotype III (2 clades closely related to Brazilian isolates)		DENV-2 strains fell into two distinct clades within the American/Asian genotype. DENV-3 strains were genotype III, and several 2006 isolates differed notably from earlier isolates. The introduction of these new DENV-2 and DENV-3 clades likely produced a shift of dominant serotype from DENV-3 to DENV-2 in 2005, and from DENV-3 to DENV-2 in 2006, possibly causing DENV-2 and DENV-3 epidemics in those years

Source: first author, year [Ref]	Region/ geographical area	Study type	Study design	Data period: date range/ year	If study:				Summary of data presentation or results/conclusion
					No. patients/ population studied (M:F)	Diagnosis (DF/DHF/ DSS etc.)	Serotype/ genotype (lineage)	Age range	
			CENTRI-SEP COLUMNS (Princeton Separations)						
Aquino 2009 [39]	Brazil (Porto Velho–RO), Colombia	Viral sequencing	Comparative: D3BR_PV7_03 strain, isolated in C6/36 cells from the serum sample, had the E and NS1 genes, and the 39UTR region sequenced Sequence analyses were then aligned with those performed previously worldwide	2003	DENV-3 strain from serum of patient with fatal DHF (N=1) and worldwide DENV-3 sequences from GenBank	DHF	DENV-3, genotype V		Phylogenetic analysis showed that DENV-3 genotype V viruses isolated in Brazil and Colombia were closely related to DENV-3 viruses isolated in Asia more than two decades ago
Añez 2011 [69]	Central America (Belize, Costa Rica, El Salvador, Guatemala, Honduras, Mexico, Nicaragua)	Gene sequencing and phylogenetic analysis	Comparative: detailed study of the phylogenetic relationships of DENV-2 from Central America (most from Nicaragua); report on the first fully sequenced DENV-2 strain from Guatemala Sequencing method: the entire viral genome of the second passage in C6/36 cells of the isolate was	1999–2009	One isolate	DF	DENV-2, American/Asian genotype (at least two lineages; clades 2a and 2b)		First report of the phylogeny, molecular clock and selection pressure analysis of DENV-2 in this region. First complete genomic sequence of a Guatemalan DENV strain, and first description from the region of codons subject to positive selection pressure in the DENV genes encoding C, E, NS2A, NS3, and NS5 proteins; some of these codons have not been described previously in any DENV-2 genotype

Source: first author, year [Ref]	Region/ geographical area	Study type	Study design	Data period: date range/ year	If study:				Summary of data presentation or results/conclusion
					No. patients/ population studied (M:F)	Diagnosis (DF/DHF/ DSS etc.)	Serotype/ genotype (lineage)	Age range	
			amplified by RT-PCR and DENV-specific forward and reverse primers, generating 11 overlapping fragments that covered the full genome. The purified PCR products were sequenced using the BigDye Terminator chemistry version 3.1 (Applied Biosystems). The sequences of overlapping fragments were assembled, evaluated and annotated using the software Sequencher, version 4.8						
Añez, 2012 [76]	Puerto Rico and Key West, Florida, USA	qRT-PCR, sequencing and phylogenetic analyses	DENV RNA was confirmed using qRT-PCR Sequencing method: extracted viral RNA was subjected to RT-PCR by using DENV-specific primers	2010 epi-demic	Six plasma samples from donors infected with DENV but asymptomatic at the time of collection	N/A	Three DENV-1 and two DENV-4 strains isolated from Puerto Rico and a DENV-1 strain from Key West, Florida, USA	N/A	Puerto Rico DENV-1 strains constitute a new lineage within genotype V different from those that circulated in Puerto Rico during the previous two decades. The newer Puerto Rico DENV-1 strains associated with strains from the Caribbean and South America. DENV-4 isolates of

Source: first author, year [Ref]	Region/ geographical area	Study type	Study design	Data period: date range/ year	If study:				Summary of data presentation or results/conclusion
					No. patients/ population studied (M:F)	Diagnosis (DF/DHF/ DSS etc.)	Serotype/ genotype (lineage)	Age range	
			to amplify the DENV structural genes region (C-prM E). In brief, fragments of approximately 3,700 and 3,500 nucleotides for DENV-1 and DENV-4, respectively, were generated by using the SuperScript III First-Strand Synthesis System (Invitrogen, Carlsbad, CA), LA Taq polymerase (Takara, Otsu, Japan), and specific DENV-1 and DENV-4 primers. Phylo-genetic analyses were conducted with E-gene sequences (1,485 nucleotides) in datasets containing a total of 36						genotype II associated with strains that have circulated in Puerto Rico throughout the 1980s and 1990s and with strains from the Caribbean region and Central America

Source: first author, year [Ref]	Region/ geographical area	Study type	Study design	Data period: date range/ year	If study:				Summary of data presentation or results/conclusion
					No. patients/ population studied (M:F)	Diagnosis (DF/DHF/ DSS etc.)	Serotype/ genotype (lineage)	Age range	
			DENV-1 and 30 DENV-4 strains						
Anzai 2004 [77]	Dominican Republic (Invivienda District, Santo Domingo City)	Nucleotide sequencing and phylogenetic analysis	Comparative: three DEN-2 virus strains, one isolated from a DHF patient and two from DF patients were compared with other strains belonging to native American and South-East Asian genotypes Sequencing method: RNA was extracted from the viral stock. Synthetic oligonucleotide primer pairs for RT-PCR were designed to amplify overlapping fragments of 500–700 bp spanning the complete DEN-2 genome. PCR products purified using Centri-Sep columns (Princeton separations, Inc.) were directly sequenced using	2001	Paediatric patients with DF or DHF (N=3)	DF; DHF	DENV-2, Asian genotype, American-Asian subtype	DF: female, aged 11 y; male, aged 6 y<br>DHF: male, aged 9 y	First genomic characterisation of DENV-2 strains from the Dominican Republic; all had extensive homology with DENV-2 from Martinique, French West Indies and Jamaica. These strains showed 26 amino acid changes that differed from both the South-East-Asian and native-American genotypes. No amino acid differences were observed between strains isolated from DF and DHF patients

Source: first author, year [Ref]	Region/ geographical area	Study type	Study design	Data period: date range/ year	If study:				Summary of data presentation or results/conclusion
					No. patients/ population studied (M:F)	Diagnosis (DF/DHF/ DSS etc.)	Serotype/ genotype (lineage)	Age range	
			the BigDye Terminator Cycle Sequencing Ready Reaction kit and an ABI Prism A310 sequence analyser						
Avilés 2002 [31]	Argentina (9 de Julio, Clorinda, Eldorado, Libertad, Puerto Esperanza, San Pedro, Wanda), Paraguay (Areguá, Asunción, Caaguazú, Capiatá, Ciudad del Este, Fernando de la Mora, Lambaré, San Lorenzo)	Phylogenetic analysis and genomic sequencing	Comparative: analysis of the genomic sequences from the C-prM and the E-NS protein 1 regions of DENV-1 from a Paraguayan isolate from 1988, plus 12 Argentinean and 11 Paraguayan isolates from 2000 were compared with published sequences of DEN-1 isolated from other countries Sequencing method: RT-PCR amplification. Purified PCR products were sequenced using the BigDye Terminator Cycle	1988–2000	DENV-1 isolates from Argentina (n=12) and Paraguay (n=11) from 2000, plus Paraguay (n=1) from 1988		DENV-1 (clade I and II)		First analysis of the genetic variability of DENV-1 viruses responsible for outbreaks in Argentina and Paraguay. All viruses belonged to the same genotype, but showed some variability and grouped in two different clades. Findings suggest that the recent epidemics in Argentina and Paraguay were not due to the introduction of a new genotype, but rather to the re-emergence of a previously circulating strain, or one that was circulating unnoticed

Source: first author, year [Ref]	Region/ geographical area	Study type	Study design	Data period: date range/ year	If study:				Summary of data presentation or results/conclusion
					No. patients/ population studied (M:F)	Diagnosis (DF/DHF/ DSS etc.)	Serotype/ genotype (lineage)	Age range	
			Sequencing Kit (Applied Biosystems, Foster City, CA) and analysed on the ABI PRISM 310 Genetic Analyzer. Amplifying primers were also used for sequencing						
Avilés 2003 [30]	Argentina (Eldorado, Libertad, San Pedro, Wanda) Paraguay (Asunción, Lambaré)	Coding/ sequencing	Comparative: the viral isolates were identified as DENV-1 by indirect immuno-fluorescence test and by PCR technique. Complete coding sequencing of six DENV-1 isolated from Paraguay and Argentina during outbreaks in 2000 Sequencing method: viral RNA was extracted from the infected gene. Amplification was by RT-PCR. Purified PCR products were directly	2000	DENV-1 isolates from patients with DF from Argentina or Paraguay (N=6)		DENV-1, American–African genotype (clade I and II)	Argentina: male (n=4) Paraguay: female (n=1), male (n=1)	The six DENV-1 strains from Argentina and Paraguay group into two different clades of the 'American-African' DENV-1 genotype; one clade is most closely related to strains isolated from Brazil in 1997, the other to a Peruvian strain isolated in 1991

Source: first author, year [Ref]	Region/ geographical area	Study type	Study design	Data period: date range/ year	If study:				Summary of data presentation or results/conclusion
					No. patients/ population studied (M:F)	Diagnosis (DF/DHF/ DSS etc.)	Serotype/ genotype (lineage)	Age range	
			sequenced using the BigDye Terminator cycle sequencing kit (Applied Biosystems, Foster City, CA)						
Barrero 2004 [32]	Argentina (Buenos Aires)	Phylogenetic analysis and sequencing	Open: DF-compatible cases in Buenos Aires City in patients who had travelled to Paraguay in 1999 and 2000 were identified and blood samples taken. All samples proved positive for DENV-1 by RT-PCR from plasma as well as from cell culture supernatant Sequencing method: RT-PCRs for the structural genes and part of NS1 obtained directly from plasma were cloned into pGemT vectors	1999 and 2000	DENV-1 isolates from patients who travelled to Paraguay in 1999 and 2000 (N=5)	Dengue fever syndrome	DENV-1, genotype V (clades I and II)		Phylogenetic analysis split Buenos Aires isolates into two clusters within American DENV-1 genotype V. Clade I was phylogenetically linked to Brazilian samples and clade II to samples from Paraguay and north-eastern Argentina. No evidence of recombination was detected
Barrero 2008 [47]	Argentina (Buenos Aires)	Genetic analysis and phylogenetic inference	Open. structural proteins C, prM/M, E and NS proteins 1	Jan–Sep 2007	Febrile patients with a history of recent travel to	DF: Classi-fication of cases:	DENV-3, genotype III	6 m–79 y (median 31 y).	First report of DENV-3 genetic characterisation in Argentina. 32/100 (32%) confirmed

Source: first author, year [Ref]	Region/ geographical area	Study type	Study design	Data period: date range/ year	If study:				Summary of data presentation or results/conclusion
					No. patients/ population studied (M:F)	Diagnosis (DF/DHF/ DSS etc.)	Serotype/ genotype (lineage)	Age range	
			and 2 from eight viruses were genetically characterised. Phylogenetic inference was performed for the E-protein and all viruses clustered with DENV-3 genotype III. Sera were obtained 0–6 days after the onset of symptoms from febrile patients with a history of recent travel to Paraguay or Brazil Sequencing method: amplicons were purified, quantified and labelled with DyET Terminator Kit. The reaction products were detected on a MegaBACE 1000 sequencing instrument by capillary electrophoresis (MegaBACE and DYEnamic ET		Brazil or Paraguay (N=100)<br><br>1:1;	non-dengue (n=55), dengue-like (n=13), classic DF (n=32)			dengue infection (both PCR- and cell culture-positive [n=18] and both PCR- and IgM-positive [n=14]. According to the IgM/IgG ratio, laboratory-confirmed cases were further classified as primary (n=21; 54%) or secondary (n=16; 41%) dengue infections. Viremic samples: 35/37 positive for DENV-3, 1/37 positive for DENV-2, and 1/37 positive for DENV-4, with a recent history of travel to Paraguay/Brazil, Costa Rica or Venezuela, respectively. Phylogenetic inference using a virus sample subset (n=8) clustered with DENV-3 genotype III.

Source: first author, year [Ref]	Region/ geographical area	Study type	Study design	Data period: date range/ year	If study:				Summary of data presentation or results/conclusion
					No. patients/ population studied (M:F)	Diagnosis (DF/DHF/ DSS etc.)	Serotype/ genotype (lineage)	Age range	
			DyeTerminator Cycle Sequencing Kit for MegaBACE						
Brown 2011 [73]	Jamaica	DENV serotyping	Open: the DENV serotypes were determined in 770 serum samples (dengue IgM antibody positive, n=469; dengue IgM negative, n=185; dengue antibody negative, n=116) taken from patients with suspected dengue who presented during (n=150) or after (n=620) the acute phase of the illness. Serotyping and antibody/RNA analysis using ELISA and RT-PCR performed on serum and cell culture supernatants of C6/36 mosquito cells inoculated with acute phase serum (n=150)	2003–2007	Patients with suspected dengue infection who presented during (n=150) or after (n=620) the acute phase of the illness Serum samples (N=770): IgM-positive (n=469); IgM-negative, (n=185); antibody-negative (n=116)	DF or non-dengue fevers plus 1 case of DHF in 2007 (DENV-2)	DENV-1 DENV-2 DENV-3 DENV-4	8 m–64 y.	20/770 (2.6%) confirmed dengue infection; male:female: 1:1; . All four serotypes were identified over the 5-year period: DENV-1 (3/20, 15%), DENV-2 (7/20, 35%), DENV-3 (3/20, 15%), DENV-4 (7/20, 35%). DENV-1, -2 and -4 were present during 2007. DENV-2 and -4 were the likely cause of the 2007−08 outbreak in Jamaica. The three strains of DENV-3 were isolated from infants aged <3 years with primary infection during 2006
Brown 2011 [74]	Jamaica		Dengue antibodies were	2003–2007	DENV serotypes	Samples from	All four serotypes were	N/A	41% of acute phase sera and 66% of post-acute sera were

Source: first author, year [Ref]	Region/ geographical area	Study type	Study design	Data period: date range/ year	If study:				Summary of data presentation or results/conclusion
					No. patients/ population studied (M:F)	Diagnosis (DF/DHF/ DSS etc.)	Serotype/ genotype (lineage)	Age range	
			detected by ELISA and DENV RNA by RT-PCR performed on serum and cell culture supernatants of C6/36 mosquito cells inoculated with acute phase serum. performed in a Perkin Elmer model 9700 thermalcycler (Applied Biosystems, Foster City, CA). The PCR products were detected by agarose gel electrophoresis in lxTris-borate buffer pH8.0 (54g trizmabase, 27.5g boric acid, 0.5MEDTA pH8.0/L) using a 2% agarose gel containing ethidium bromide (0.5/g/mL; Sigma Chemic St Louis, Mo) at 100V for 1h then visualized on a		were determined in 770 serum samples selected consecutively from a cohort of 2,248 patients with dengue-like illnesses	dengue IgM antibody-positive (n=469), dengue IgM-negative (n=185) and dengue antibody-negative (n=116) patients with suspected dengue	identified over the five-year period: DENV-1 (15%), DENV-2 (35%), DENV-3 (15%) and DENV-4 (35%)		from patients with current primary or secondary dengue; 41% and 35% of acute and post-acute phase sera, were from patients with secondary dengue or past exposure only. DENV RNA was found in 20/770 samples (2.6%). Only1.5% (9/620) of sera collected after the acute phase of illness tested positive for DENV RNA compared with 2.6% (4/150) of sera collected during the acute phase and 7.3% of cell culture supernatants inoculated with acute phase serum (11/150, p=0.001). The results confirm that DENV-2 and DENV-4 were the likely causative viruses of the 2007–2008 dengue outbreak in Jamaica. This study highlights the increasing threat of dengue and severe dengue disease to the Jamaican population. Preventative measures including laboratory surveillance and vector control should be strictly maintained at the highest level

Source: first author, year [Ref]	Region/ geographical area	Study type	Study design	Data period: date range/ year	If study:				Summary of data presentation or results/conclusion
					No. patients/ population studied (M:F)	Diagnosis (DF/DHF/ DSS etc.)	Serotype/ genotype (lineage)	Age range	
			UV trans-illuminator and photographed						
Campos 2013 [50]	Brazil (Rio de Janeiro)	Phylogenetic analyses	Phylogenetic analysis of DENV-4 strains. The multiple nucleotide sequence alignment was analysed by the Markov Chain Monte Carlo method implemented in the program MrBayes (version 3.0) applying the GTR substitution model	2011		DF	DENV-4, genotype IIb DENV-4, genotype I		Infections serotyped by Rio de Janeiro State Laboratory during 2011 outbreak: 32% DENV-1, with majority of other infections caused by DENV-4. Phylogenetic analysis of DENV-4 strains (n=10) revealed the presence of DENV-4 genotype II b; these strains are closely related to those detected in the city of Roraima in 2010 (strain Br246RR) and the state of São Paulo State in 2011 (strain SPH317947), and strains from Venezuela and Colombia. One characterised strain (RJ1243581) clustered with DENV-4 genotype I and is closely related with strains AM1619 and AM750 from the city of Manaus
Carrillo-Valenzo 2010 [67]	Mexico (15 states: Baja California Sur, Chiapas, Estado de Mexico, Guanajuato, Jalisco, Morelos, Nuevo León, Oaxaca, Queretaro, Quintana Roo, Sinaloa, Sonora, Tabasco, Tamaulipas, Veracruz)	Phylogenetic analysis and sequencing	Open: examination of patterns of sequence evolution in 83 E-gene sequences using immuno-fluorescence or RT-PCR Sequencing method: E-gene sequencing was	1980–2007	E-gene sequences from DENV isolates from patient sera or GenBank (N=83): DENV-1 (n=23), DENV-2 (n=37), DENV-3 (n=10), DENV-4 (n=13)	DF	DENV-1, genotype III (three distinct lineages) DENV-2, American genotype DENV-2, Cosmopolitan genotype DENV-2, Asian/American genotype		Multiple introductions of DENV viral lineages but strikingly little co-circulation. DENV evolution in Mexico is typified by frequent lineage replacement, involving members of the same viral genotype. A replacement event involving different genotypes was observed with DENV-2, and viral lineages that are new to Mexico are

Source: first author, year [Ref]	Region/ geographical area	Study type	Study design	Data period: date range/ year	If study:				Summary of data presentation or results/conclusion
					No. patients/ population studied (M:F)	Diagnosis (DF/DHF/ DSS etc.)	Serotype/ genotype (lineage)	Age range	
			performed as described previously (Roca 2009 [57]). Amplicons for direct sequencing were generated by PCR for the entire E-gene				DENV-3, genotype III (three lineages) DENV-4, genotype II (two lineages)		described for DENV-1, DENV-3 and DENV-4
Cisneros 2006 [70]	Mexico (Oaxaca: cities of Huatulco, Juchitan, Salina Cruz, Tonala, Tuxtepex)	Phylogenetic analysis and sequencing	Open: the nucleotide sequence of the C and a portion of the prM protein genes of 8 DENV-2 isolated from acute-phase plasma from patients with DF and DHF from the 2000/2001 epidemic were sequenced Sequencing method: a fragment with the expected size of 594 bp (23 bp of the UTR-5 region, nucleotide 73–96, the structural C protein gene and nucleotides 438–572 of the prM gene) was amplified by RT-	2001	DENV-2 isolates from acute-phase plasma of patients with DF/DHF (N=8)	DF/DHF: 1:1	DENV-2, American/Asian genotype		DENV-2 isolates were of the American/Asian genotype and were most similar to the Jamaica and Venezuelan isolates MARA3, LARD1996 and LARD1910. DENV-2 strains of American/Asian genotype, probably from South-East Asia, are circulating in Oaxaca. All of these genotypes have the potential to cause DHF, independently of the host or environment

Source: first author, year [Ref]	Region/ geographical area	Study type	Study design	Data period: date range/ year	If study:				Summary of data presentation or results/conclusion
					No. patients/ population studied (M:F)	Diagnosis (DF/DHF/ DSS etc.)	Serotype/ genotype (lineage)	Age range	
			PCR using Super Script^{TM} One-Step RT-PCR with Platinum R Taq. For automated sequencing, spin column-purified (Quiagen, Chatsworth, Calif.) DNA fragments were analysed by the cycle-sequencing dye terminator method. BigDye Terminator Cycle Sequencing Ready Reaction kit						
Cruz 2010 [54]	Peru (including provinces of Amazonas, Cajamarca, Huánuco, Junín, La Libertad, Lambayeque, Loreto, Madre de Dios, Piura, San Martin, Tumbes, Ucayali), Ecuador	Phylogenetic analysis and sequencing	Comparative: DENV-2 was obtained from acute-phase sera collected from patients enrolled in the febrile surveillance programme from 1995 to 2009. Sequences generated from E/NS1 gene junction and ENV gen. were compared to global	1995–2009	DENV-2 isolates from acute-phase sera from patients in Peru (n=41) or Ecuador (n=5)		DENV-2, American genotype DENV-2, American/Asian genotype		American and American/Asian DENV-2 genotypes co-circulated during the Peruvian north-western outbreak in 2000, with the former disappearing from 2001 onwards. Both genotypes were similar to those isolated in Ecuador during a dengue outbreak prior to 2000. American/Asian genotypes circulating from 2002–09 in the Amazon region were more closely related to Brazilian DENV-2 strains. Peruvian DENV-2 American/Asian genotypes fell into two clades, formed by

Source: first author, year [Ref]	Region/ geographical area	Study type	Study design	Data period: date range/ year	If study:				Summary of data presentation or results/conclusion
					No. patients/ population studied (M:F)	Diagnosis (DF/DHF/ DSS etc.)	Serotype/ genotype (lineage)	Age range	
			sequences obtained from GenBank Sequencing method: viral RNA (5 from Ecuador and 41 from Peru) extracted using the QIAamp Viral RNA Mini Kit (QIAGEN) and amplified using RT-PCR. RT-PCR products from E/NS1 and E regions were sequenced directly using the BigDye sequencing kit						isolations from 2000–09 and 2009. DENV-2 strain sequences from Madre de Dios during the 2009 outbreak grouped in different clades and showed a temporal circulation of genetically different viruses in the Amazon region. The same American/Asian genotype clades previously identified in Paraguay and Brazil were also found to be circulating in Peru, but unlike in those countries, the DENV-2 virus was not displaced by DENV-3 in 2009
Cruz 2013 [55]	Bolivia, Ecuador, Paraguay, Peru	Phylogenetic analysis and sequencing	Comparative: the E-gene region of DENV-2 isolates was sequenced. Sequences were aligned and compared to a global sample of DENV-2 viruses Sequencing method: RT-PCR was performed to amplify the entire 1485 bp E-gene. Amplicons were purified with Centri-Sep	2000–2012	DENV-2 isolates from acute-phase sera (N=56): Bolivia (n=11), Ecuador (n=2), Paraguay (n=3), Peru (n=40: north-west [Piura and Tumbes, n=9], north-eastern Amazon basin [Loreto, n=17], eastern Amazon basin [Ucayali, n=3],		DENV-2, American genotype DENV-2, American/ Asian genotype (lineage I, clades A and B) DENV-2, American/ Asian genotype (lineage II, clades E and F)		The DENV-2 American and American/Asian genotypes were found in Peru; the former appeared to become extinct after 2000, to be replaced by the latter. The emergence of the American/Asian genotype coincided with an increase in disease severity. DENV-2 American/Asian genotype sequences from Peru are divided into two clearly defined lineages, with lineage II replacing lineage I after 2009. Since 2000, the evolution of DENV-2 American/Asian genotype in

Source: first author, year [Ref]	Region/ geographical area	Study type	Study design	Data period: date range/ year	If study:				Summary of data presentation or results/conclusion
					No. patients/ population studied (M:F)	Diagnosis (DF/DHF/ DSS etc.)	Serotype/ genotype (lineage)	Age range	
			columns (Invitrogen) and sequenced directly using the BigDye Terminator sequencing kit version 3.1 (Applied Biosystems)		south-eastern Amazon basin [Madre de Dios, n=10], Junin, n=1)				Peru can be characterised by the introduction of four different clades within lineages I and II. Lineages I and II were both independently introduced into north-western Peru (via Ecuador, Colombia, and/or Venezuela; lineage I clade A in 2000, lineage II clade E in 2011) and eastern Peru (via Brazil and/or Bolivia; lineage I clade B in 2002, lineage II clade F in 2009). Lineage II clade F is distinct from those previously circulating in the region, and a particularly virulent strain that is associated with the large DHF epidemic in 2010
de Araújo 2009 [40]	30 countries worldwide (Bangladesh, Bolivia, Brazil, China, Cuba, East Timor, Ecuador, Fiji, French Polynesia, India, Indonesia, Japan, Malaysia, Martinique, Mexico, Myanmar, Nicaragua, Panama, Paraguay, Peru, Philippines, Puerto Rico, Singapore, Somalia, Sri Lanka, Tahiti, Taiwan, Thailand,	Phylogenetic analysis and sequencing	Open: retrieved and analysed the full-length (1,479 bp) and partial (822 bp) E-gene sequences of 103 DENV-3 strains from 30 different countries around the world, representative of all known genotypes. Tree reconstructions were performed by the Neighbour-	None given	DENV-3 E-gene sequences from GenBank (N=103)		DENV-3, genotype I DENV-3, genotype II DENV-3, genotype III DENV-3, genotype IV DENV-3, genotype V		Phylogenetic analysis of DENV-3 sequences isolated in Brazil and Colombia confirmed their classification as GV. An unpublished DENV-3 E sequence with a high similarity score to the GV strains, deposited in the GenBank database in 2006, corresponded to a virus isolated in state of Pará, Brazil, in 1989. This contrasts with official records that DENV-3 was first isolated in Brazil from an autochthonous case in 2000. The GV Brazilian strains were also unexpectedly similar to the prototype DENV-3 strain

Source: first author, year [Ref]	Region/ geographical area	Study type	Study design	Data period: date range/ year	If study:				Summary of data presentation or results/conclusion
					No. patients/ population studied (M:F)	Diagnosis (DF/DHF/ DSS etc.)	Serotype/ genotype (lineage)	Age range	
	Venezuela, Vietnam)		Joining method in 1,000 bootstrapped data sets						identified in the Philippines in 1956
de Araújo 2009 [A]	31 countries worldwide	Phylogenetic analysis	Open: viral isolation, followed by viral RNA extraction, amplification and sequencing of E-gene region, phylogenetic analysis, and analysis of spatio-temporal dispersion pattern Sequencing method: the complete E-gene (1,479 bp in length) was amplified by RT-PCR. Amplicons were directly sequenced in both directions using a BigDye Terminator Cycle Sequencing Ready Reaction kit (Applied Biosystems, US)	1956–2006	DENV-3 E sequences from GenBank (N=200)		DENV-3, genotype I DENV-3, genotype II DENV-3, genotype III DENV-3, genotype IV DENV-3, genotype V		Phylogenetic analysis revealed a clear geographical subdivision of DENV-3 strains. Strains more recently isolated in the Americas (1994–2006) segregated into distinct monophyletic clusters within the main genotypes, indicating formation of a geographically distinct, mostly self-contained region with regard to DENV-3 viruses, with few instances of repeated gene flow. Phylogeographic analysis revealed that the co-circulation of different DENV-3 genotypes in a single location is a rare event
de Araújo 2012 [46]	Brazil (states of Espirito Santo, Goias, Rio de Janeiro), comparing with 29 countries worldwide	Reconstruc-tion of the spatio-temporal dispersion pattern of	Open: viral isolation, followed by viral RNA extraction, amplification and sequencing of E-	1981–2009	Brazilian patients with confirmed DENV-3 infection (N=19): from		DENV-3, genotype III<br><br>GenBank DENV-3 E-gene sequences:		At least four introductions of the same DENV-3 genotype III in Brazil; only two viral lineages seem to have become established and disseminated. The Caribbean

Source: first author, year [Ref]	Region/ geographical area	Study type	Study design	Data period: date range/ year	If study:				Summary of data presentation or results/conclusion
					No. patients/ population studied (M:F)	Diagnosis (DF/DHF/ DSS etc.)	Serotype/ genotype (lineage)	Age range	
		DENV-3 lineage circulating in Brazil and the Americas	gene region, phylogenetic analysis, and analysis of spatio-temporal dispersion pattern Sequencing method: the complete E-gene (1,479 bp in length) was then amplified by RT-PCR. Amplicons were directly sequenced in both directions using a BigDye Terminator Cycle Sequencing Ready Reaction kit (Applied Biosystems, US)		states of Espirito Santo (n =3), Goias (n =4), Rio de Janeiro (n=12) states		from Brazil in 2001–2009 (n=107), 29 countries worldwide in 1981–2009 (n=457)		islands were the main source of the DENV-3 viruses, and the northern and south-eastern Brazilian regions seem to be the most important hubs of introduction and dissemination.DENV-3 strains circulated for ≥ 1–2 years until they met favourable conditions for the initiation of an outbreak
de Castro 2013 [38]	Brazil (Rio de Janeiro)	Phylogenetic and vector-virus-human host	Open: sequencing of the entire genome of one DENV-3 isolate from *Aedes aegypti* (n=4) and naturally infected human hosts (n=10) from Rio de Janeiro between 2001 and 2008; characterisation of the 3′ UTR in comparison with	2001–2008	DENV-3 isolates from *Aedes aegypti* (n=4) and naturally infected human hosts (n=10)		DENV-3, genotype III		Based on analysis of the complete genome and 3′ UTR, DENV-3 isolated from both vector and human host was genotype III. The majority of DENV-3 isolates were characterised by an 11-nucleotide insertion in the 3′ UTR, although strains carrying an 8-nucleotide deletion, or a substitution leading to stop codon formation, were also observed

Source: first author, year [Ref]	Region/ geographical area	Study type	Study design	Data period: date range/ year	If study:				Summary of data presentation or results/conclusion
					No. patients/ population studied (M:F)	Diagnosis (DF/DHF/ DSS etc.)	Serotype/ genotype (lineage)	Age range	
			strains isolated from naturally infected mosquitoes and humans Sequencing method: viral RNA was extracted using QIAamp Viral RNA Mini kit (Qiagen). After RT-PCR amplification, PCR products were sequenced in both directions using the BigDye Dideoxy Terminator sequencing kit (Applied Biosystems)						
Figueiredo 2008 [51]	Brazil (Manaus)	Serological and molecular characteris-ation	Open: all serum samples collected during acute phase of illness and tested for DENV infection by three methods: virus culture; detection of IgM antibodies to DENV by an ELISA on serum samples from patients >7 days after onset of symptoms;	January 2005 to June 2007	DENV-4 isolates from patients at a tropical medicine centre (N=3)		DENV-4<br>DENV-4 co-infected with DENV-3 (1 sample)		DENV-4 was detected in 3 samples by virus culture or RT-PCR, either as a single infection (n=2) or as a co-infecting virus with DENV-3 (n=1). Patients had no travel history, indicating that DENV-4 was autochthonous in Manaus

Source: first author, year [Ref]	Region/ geographical area	Study type	Study design	Data period: date range/ year	If study:				Summary of data presentation or results/conclusion
					No. patients/ population studied (M:F)	Diagnosis (DF/DHF/ DSS etc.)	Serotype/ genotype (lineage)	Age range	
			nucleic acid amplification and typing by RT–PCR Sequencing method: amplicons were cloned into a TA vector (Invitrogen), and >3 colonies for each sample were sequenced in both directions by using the BigDye Terminator Cycle Sequence Kit						
de Mora 2009 [62]	Ecuador, comparing with 11 other Latin American countries and 20 other countries worldwide	Phylogenetic analysis and sequencing	Open: nine serum samples from 23 Ecuadorian patients with dengue-like syndromes were found to be DENV-3. Full-length E-gene nucleotide sequences, corresponding to position 1,014 through 2,413 of the DENV genome, were obtained from these nine patients, and aligned with 48	None given	DENV-3 genotype III sequences from patient isolates (n=9) and GenBank (Latin America [n=48], elsewhere [n=20])	DF	DENV-3 genotype III (at least 6 clades)		At least 6 different DENV-3 genotype III clades were observed. Amino acids substitutions were found in domain III E protein neutralisation epitopes and in surface-exposed domain II and III E protein amino acid sequences

Source: first author, year [Ref]	Region/ geographical area	Study type	Study design	Data period: date range/ year	If study:				Summary of data presentation or results/conclusion
					No. patients/ population studied (M:F)	Diagnosis (DF/DHF/ DSS etc.)	Serotype/ genotype (lineage)	Age range	
			comparable sequences of DENV-3 from strains isolated in 11 different Latin American countries, as well as with 20 sequences from all DENV-3 genotypes isolated elsewhere Sequencing method: samples underwent RT-PCR and amplicons were purified using a QIAquick PCR Purification Kit from QIAGEN. The sequence reaction was carried out using the BigDye DNA sequencing kit (Perkin–Elmer) on a 373 DNA sequencer apparatus (Perkin–Elmer)						
de Souza 2011 [52]	Brazil (states of Parana, Rio Grande do Sul, São Paulo)	Bayesian phylogenetic analysis and sequencing	Comparative: nucleotide sequences of the E-gene were determined and compared with sequences	Feb–March 2011	Isolates from patient sera of autochthonous strains of DENV-4 (number not given)		DENV-4, genotype II		All DENV-4 samples were genotype II, and closely related to strains circulating since 1981 in South America, but having undergone recent evolution for ≥4–6 years. DENV-4 may have

Source: first author, year [Ref]	Region/ geographical area	Study type	Study design	Data period: date range/ year	If study:				Summary of data presentation or results/conclusion
					No. patients/ population studied (M:F)	Diagnosis (DF/DHF/ DSS etc.)	Serotype/ genotype (lineage)	Age range	
			representative of genotypes I, II, III and sylvatic for DENV-4 retrieved from GenBank. All new DENV-4 strains characterised in this study were isolated directly from patient serum and detected by RT-PCR Sequencing method: total RNA was extracted from infected cells. RT-PCR products were purified and directly sequenced using the BigDye v.3.1 Terminator chemistry. Sequences were determined using the Applied Biosystems 3130XL DNA sequencer						penetrated the Brazilian population earlier than 2010, being present but not detected, due to a higher prevalence of DENV-1 and DENV-2, and the failure of the surveillance system to identify the milder disease commonly associated with DENV-4
dos Santos 2011 [25]	Brazil (Rio de Janeiro state)	Phylogenetic analysis and sequencing	Open: DENV-1 strains (n=10; from 1986 [n=2], 2009 [n=3], 2010	None given, but likely late	DENV-1 E-gene sequences from two		DENV-1 genotype V (America/Africa)		First report of multiple DENV-1 lineages circulating in Brazil. DENV-1 isolated during 2009–10 belonged to

Source: first author, year [Ref]	Region/ geographical area	Study type	Study design	Data period: date range/ year	If study:				Summary of data presentation or results/conclusion
					No. patients/ population studied (M:F)	Diagnosis (DF/DHF/ DSS etc.)	Serotype/ genotype (lineage)	Age range	
			[n=4] and 2011 [n=1]) extracted directly from serum previously detected by RT-PCR or originally isolated from cell culture. Viral RNA was extracted from infected cell culture supernatant or directly from the patients' serum for RT-PCR and sequencing Sequencing method: amplification of the C/prM/M/E region of 2,325 bp. Sequencing reactions performed as recommended in the BigDye Dideoxy Terminator sequencing kit (Applied Biosystems) and were analysed using an automated 3130 DNA Sequencer	2010–2011	epidemiol-ogically distinct periods: 1986 (n=2), 2009–2011 (n=8)		(three distinct clades)		genotype V (Americas/Africa), but grouped in a distinct clade (lineage II) to that of earlier DENV-1 isolates (lineage I). However, strains isolated in 2011 grouped together to form another distinct clade (lineage III)
Drumond 2012 [29]	Brazil (São José do Rio Preto, São Paulo state)	Phylogenetic analysis and sequencing	Open: different lineages of 59 complete	2008	Genomic sequences from DENV-1		DENV-1, genotype V (three lineages)		All isolates belonged to genotype V and are subdivided into three

Source: first author, year [Ref]	Region/ geographical area	Study type	Study design	Data period: date range/ year	If study:				Summary of data presentation or results/conclusion
					No. patients/ population studied (M:F)	Diagnosis (DF/DHF/ DSS etc.)	Serotype/ genotype (lineage)	Age range	
			genome sequences were defined based on the branching patterns of the phylogenetic tree (supported by bootstrap values C98%) Sequencing method: viral cDNA was amplified by PCR; 96 amplicons, each 500–900 nucleotides in length, were bidirectionally sequenced using the Big Dye chemistry on ABI3730xl DNA sequencers		isolates from serum of patient diagnosed with DF (n=1), and from GenBank (n=59)				lineages, which were introduced during four different events (1984–85, 1997–99, and two events in 2004–07). The introduction of new strains resulted in lineage replacement and an increase in DENV-1 genetic diversity, but not positive selection<br>DENV-1 dynamics in Brazil characterised by co-circulation and generation of genetically distinct viruses as a result of local evolution, or exogenous virus introduction
Drumond 2013 [36]	Brazil (São José do Rio Preto, São Paulo state)	Phylogenetic analysis and sequencing	Open: the whole ORF or E-sequences were used to perform phylogenetic, phylogeographic and evolutionary analyses. Isolates from São José do Rio Preto/São Paulo were grouped within one lineage (BR3) close to isolates	2008	Genomic sequences from DENV-2 isolates from sera of patients diagnosed with DF (n=12), and from GenBank (n=79)	DF	DENV-2, American/Asian genotype (three distinct lineages)		DENV-2 isolates clustered in the BR3 lineage with two Brazilian strains from the northern region and a strain isolated in Jamaica in 2007. All DENV-2 isolates grouped within the American/Asian genotype, together with isolates from South and Central America and the Caribbean, as previously demonstrated for other Brazilian isolates

Source: first author, year [Ref]	Region/ geographical area	Study type	Study design	Data period: date range/ year	If study:				Summary of data presentation or results/conclusion
					No. patients/ population studied (M:F)	Diagnosis (DF/DHF/ DSS etc.)	Serotype/ genotype (lineage)	Age range	
			from Rio de Janeiro, Brazil Sequencing method: PCR reactions produced 96 overlapping amplicons, each 500–900 nucleotides in length, which were subsequently sequenced bidirectionally using the Big Dye chemistry on ABI3730xl DNA sequencers						
Dussart 2006 [82]	French Guiana and the French Caribbean	Phylogenetic analysis and sequencing	Comparative. 8 DENV-4 strains isolated from human sera, 6 from French Guiana in 1993–5 and 2 from 2004–5; .also 2 human serum specimens from Martinique and 1 from Guadeloupe that were positive for DENV-4 during dengue surveillance in the fourth quarter of 2004 were tested	2004–2005, versus 1993–1995	DENV-4 isolates from patient sera (1993–1995: French Guiana, n=6; 2004–2005: French Guiana, n=2; Guadeloupe, n=1; Martinique, n=2), and DENV-4 sequences from GenBank (n=87)		DENV-4, genotype II		DENV- 4 has recently re-emerged in Martinique, Guadeloupe, and French Guiana. Phylogenetic analyses of strains isolated from 2004–5 showed that they belong to DENV-4 genotype II, but to a different cluster than strains isolated from 1993–95

Source: first author, year [Ref]	Region/ geographical area	Study type	Study design	Data period: date range/ year	If study:				Summary of data presentation or results/conclusion
					No. patients/ population studied (M:F)	Diagnosis (DF/DHF/ DSS etc.)	Serotype/ genotype (lineage)	Age range	
			Sequencing method: DENV-4 infection was confirmed by using virus isolation on AP 61 cells. A 1,940-bp region of the genome for the E-gene and adjacent prM/M and NS1 junctions were analysed. Each PCR product was cloned by using the TOPOTA Cloning kit . For each isolate, 3 clones were sequenced by Genome Express						
Faria 2013 [33]	Brazil (6 states, not specified)	Molecular characteris-ation and phylogenetic analysis	Comparative: viral strains isolated from patients presenting different disease manifestations (n=34) were sequenced and compared with reference strains. All strains were determined as DENV-2	1990–2010	Full-length or partial gene sequences from DENV-2 isolates from patients presenting with DF (n=19), DHF (n=3), DSS (n=1), and GenBank (n=22, worldwide isolates)	DF, DHF, DSS	DENV-2, Southeast Asian genotype (lineages I and II)		DENV-2 strains comprised two epidemiologically distinct groups: one represented by strains isolated from 1990–2003 (South-East Asian genotype lineage I) and one from strains isolated from 2007–10 (South-East Asian genotype lineage II). The percentage identity of the latter with a Dominican Republic strain isolated in 2001, combined with the percentage of divergence with strains first introduced

Source: first author, year [Ref]	Region/ geographical area	Study type	Study design	Data period: date range/ year	If study:				Summary of data presentation or results/conclusion
					No. patients/ population studied (M:F)	Diagnosis (DF/DHF/ DSS etc.)	Serotype/ genotype (lineage)	Age range	
			serotype by RT-PCR and/or virus isolation Sequencing method: viral RNA was extracted using QIAamp Viral RNA Mini kit (Qiagen) and subject to RT-PCR amplification. Amplification of C/prM/M/E region of 2,325 bp produced amplicons; sequencing reactions were performed as recommended in the BigDye Dideoxy Terminator sequencing kit (Applied Biosystems) and the products were analysed using an automated 3130 DNA Sequencer (Applied Biosystems)						into the country in the 1990s, suggests that these were a new viral lineage introduced from the Caribbean
Forshey 2009 [66]	Peru (north-eastern region): Guayaquil (Ecuador), Iquitos, Lima, Piura, Trujillo,	Phylogenetic analysis and sequencing	Comparative: patient sera were injected into African	2000–March 2009	E-gene sequences from DENV-4 isolates from		DENV-4 genotype II (different clades for 2000 vs		From 2000–8, DENV-3 (n=1,572 isolates) was the dominant serotype in circulation in the study sites,

Source: first author, year [Ref]	Region/ geographical area	Study type	Study design	Data period: date range/ year	If study:				Summary of data presentation or results/conclusion
					No. patients/ population studied (M:F)	Diagnosis (DF/DHF/ DSS etc.)	Serotype/ genotype (lineage)	Age range	
	Tumbes, Yurimaguas		green monkey Vero cells or *Ae. albopictus* C6/36 cells and examined for a range of arboviruses, including all 4 DENV serotypes, by immuno-fluorescent assay Sequencing method: to characterise the DENV-4 isolates, a 1,485-bp sequence covering the entire mature E-gene was amplified and sequenced (no details given) from a representative set of viruses from Guayaquil (n=6), Tumbes (n=6), Piura (n=2), Trujillo (n=1), Iquitos (n=9), Yurimaguas (n=7), and Lima (n=2), all collected during		Guayaquil (n=6), Iquitos (n=9), Lima (n=2), Piura (n=2), Trujillo (n=1), Yurimaguas (n=7)		2006–2009 isolates: 2000 strains clustered more closely with a previous 1994 Ecuador isolate, related to the initial 1981 Caribbean DENV-4 strains [designated as subtype A]; 2006–2009 isolates most closely related to recent DENV-4 isolates from Venezuela, and formed a lineage distinct from previously published DENV-4 Caribbean basin strains; this lineage is distinguished from previously reported DENV-4 genotype II strains by 3 conserved amino acid variations in the E protein: S64L, A235T, and S403A)		followed by DENV-1 (n=205 isolates) and DENV-2 (n=87 isolates). DENV-4 was rare until 2006–07.By October 2008, DENV-4 had almost completely displaced DENV-3. Phylogenetic analysis of 2008–09 isolates support their inclusion into DENV-4 genotype II, forming a lineage distinct from strains that had previously circulated in the region

Source: first author, year [Ref]	Region/ geographical area	Study type	Study design	Data period: date range/ year	If study:				Summary of data presentation or results/conclusion
					No. patients/ population studied (M:F)	Diagnosis (DF/DHF/ DSS etc.)	Serotype/ genotype (lineage)	Age range	
			2000 through 2009						
Gutierrez 2011 [71]	Nicaragua (District II of Managua)	Two parallel studies of paediatric dengue: i) hospital-based ii) community-based prospective cohort	DENV RNA detected by RT-PCR (after extraction from serum samples by using the QIAamp Viral RNA Mini Kit (Qiagen, Valencia, California); seroconversion is demonstrated by DENV-specific IgM capture ELISA antibody titre by inhibition ELISA	Hospital-based study: 1998–2010; cohort study: 2004–2010; focus on time period during 2009–2010 epi-demic	Hospital-based study (August 2009 to January 2010): N=396 Cohort study (August 2009 to June 2010): N=3,711 (Hospital-based study: 166:166 (2005–2009), 106:106 (2009–2010); Cohort study: 95:86 (2004–2009), 85:85 (2009–2010))	DF, DHF, DSS	DENV-2 DENV-3, Asian-American genotype (no changes in genotype or clade between 2008 and 2011)	Hospital-based study: 6 m–14 y, mean (SE): 7.2 (2.6) y (2004–2009), 8.2 (3.0) y (2009–2010); p=0.002 Cohort study: 2–14 y, mean (range): 8.4 (4.8–11.5) y (2004–2009), 8.6 (5.2–10.8) y (2009–2010); p=0.8831.	Hospital-based study: 212/396 (54%) confirmed dengue infection (August 2009 to January 2010); majority were DENV-3 serotype (88.8%); Cohort study: 170/3,711 (4.6%) confirmed dengue infection (August 2009 to June 2010); majority were DENV-3 serotype (83.9%); The 2009–10 dengue epidemic in Managua involved an atypical presentation, with early onset of signs of poor peripheral perfusion (DF with compensated shock). Multivariate analysis revealed only study year 2009–10 as a significant risk factor for DF with compensated shock. In 2009, the circulation of pandemic influenza A H1N1 overlapped with the dengue season in Managua. The unusual presentation of dengue may have been partly due to immunomodulation by a prior influenza H1N1-2009 infection
Huhtamo 2013 [B]	Venezuela	Molecular epidemio-logical	Open: sequence analysis was performed for 122 DENV-2 envelope gene sequences	1995–2005	E-gene sequences from DENV-2 isolates (n=23) and GenBank (n=99, of	DF, DHF	DENV-2, American-Asian genotype (6 genetic lineages)		Isolates fell into 6 genetic lineages exclusively within the American-Asian genotype, suggesting that DENV-2 has undergone genetic diversification locally

Source: first author, year [Ref]	Region/ geographical area	Study type	Study design	Data period: date range/ year	If study:				Summary of data presentation or results/conclusion
					No. patients/ population studied (M:F)	Diagnosis (DF/DHF/ DSS etc.)	Serotype/ genotype (lineage)	Age range	
			including the 23 DENV-2 strains sequenced in this study and 55 Venezuelan DENV-2 viruses for which the complete E-gene sequence was available in the GenBank database<br>Sequencing method: E-gene regions were amplified by RT–PCR and directly sequenced using a set of DENV-2 E-gene-specific primers		which n=55 from Venezuelan DENV-2 strains)				in Venezuela since the 1980s. Venezuelan DENV-2 strains were only partly temporally clustered, suggesting co-circulation of variable strains, and had some unique E-gene codon changes
Kochel 2008 [59]	Peru (Piura, Tumbes [coastal]; Iquitos, Yurimaguas [jungle cities]; Lima), Bolivia (Santa Cruz), Ecuador (Cañar, Guayas), Venezuela (Aragua)	Phylogenetic analysis and sequencing	Comparative: sequencing of C/prM/M and E-genes from 22 DENV-3 strains and comparison with other DENV-3 subtype III viruses<br>Sequencing method: PCR amplified a sequence of 2,273-bp fragment from positions 278–2,550 encompassing	2000–2005	C, prM/M, E-gene sequences from DENV-3 strains and GenBank (n=22)		DENV-3, subtype III (three clades)		All viruses isolated in 2000–5 belonged to DENV-3 genotype III, but were divided into three genetic lineages, comprising strains from Venezuela, Peru–Ecuador and Bolivia–Brazil.<br>The most recent DENV-3 viruses currently circulating in South America have evolved and form phylogenetic groups that are distinct from those of Central America

Source: first author, year [Ref]	Region/ geographical area	Study type	Study design	Data period: date range/ year	If study:				Summary of data presentation or results/conclusion
					No. patients/ population studied (M:F)	Diagnosis (DF/DHF/ DSS etc.)	Serotype/ genotype (lineage)	Age range	
			regions of part of C, prM/M and E-genes. Sequence analyses were performed on an automated Applied Biosystems 3100 Avant Genetic Analyzer DNA sequencer. Gel purified PCR products were sequenced directly using the BigDye Terminator Cycle Sequencing Kit						
Mamani 2011 [56]	Peru (Iquitos, Lima, Tarapoto, Trujillo, Yurimaguas)	Phylogenetic analysis and sequencing	Comparative: analysis of 8 samples collected during dengue surveillance and comparison with others reported in the Genbank Sequencing method: viral RNA was extracted and E/ NS1 amplicons were sequenced and analysed by phylogeny	Novem-ber 2010 to January 2011	Gene sequences from DENV-2 isolates collected during dengue surveillance and GenBank (n=8)		DENV-2 American/Asian genotype		Peruvian DENV-2 isolates from a severe dengue outbreak in 2010 were American/Asian genotype, and closely related to DENV-2 isolates circulating in Brazil during 2007–8, which were similarly associated with severe dengue cases and deaths. The 2010 isolates were genetically distinct from those circulating in Peru in 2001, which were not associated with severe disease
Mendez 2010 [53]	Colombia	Phylogenetic analysis	DENV serotype identified by	Samples collected	74 viruses obtained from	N/A	DENV-1	N/A	DENV-1 Colombian isolates belonged to the formerly

Source: first author, year [Ref]	Region/ geographical area	Study type	Study design	Data period: date range/ year	If study:				Summary of data presentation or results/conclusion
					No. patients/ population studied (M:F)	Diagnosis (DF/DHF/ DSS etc.)	Serotype/ genotype (lineage)	Age range	
			monoclonal antibodies and confirmed by RT-PCR. Cell culture supernatants were used to extract viral RNA using QIAamp Viral RNA Minikit (Qiagen) Sequencing method: amplified products (from RT-PCR or nested PCR) were purified using QIAquick PCR Purification Kit (QIAGEN, Germany) and then used as template for sequencing reactions using the ABI Prism Dye Terminator Cycle Sequencing Ready Reaction Kit (Applied Biosystems, Foster City, CA)	1978–2007	symptomatic patients were isolated in mosquito cell culture				defined genotype V; only one virus isolate was genotype I. The oldest strains were closely related to those detected for the first time in America in 1977 from the Caribbean. A split in 1987 generated two lineages that have been evolving separately
Mondini 2009 [43]	Brazil (São José do Rio Preto city, in the north-western region of São Paulo state)	Reconstruction of the spatio-temporal dispersion pattern of	Open: geographic and temporally structured phylogenetic	January to June 2006	Gene sequences from sera of patients presenting	DF, DH	DENV-3 (two lineages)		All samples were DENV-3 and related to strains circulating on Martinique in 2000–01. DENV-3 from São José do Rio Preto formed a

Source: first author, year [Ref]	Region/ geographical area	Study type	Study design	Data period: date range/ year	If study:				Summary of data presentation or results/conclusion
					No. patients/ population studied (M:F)	Diagnosis (DF/DHF/ DSS etc.)	Serotype/ genotype (lineage)	Age range	
		DENV-3 lineages	data, to examine the spread of at least 2 DENV lineages circulating in an urban area Sequencing method: viral RNA was extracted and following 2 PCRs the fragments were purified and sequenced with the BigDye v3.1 Terminator (Applied Biosystems, Foster City, CA, USA)		with dengue-like symptoms (n=82)				monophyletic group (lineage 1; n=60), closely related to the remaining isolates (lineage 2; n=22). These lineages are assumed to have appeared before 2006 on separate occasions
Nogueira 2008 [41]	Brazil (Acre, Porto Velho, Rio Branco and Rondônia, Rio de Janeiro)	Phylogenetic analysis and sequencing	Comparative: complete genome sequencing of the samples, which were then compared with other sequences Sequencing method: complete genomes were amplified by means of overlapping RT-PCR products. The amplicons were directly sequenced using	2002–2004	Genomic sequences from DENV-3 isolates from sera of patients diagnosed with DF (n=9), and from GenBank (n=114)	DF	Rondônia (2002): DENV-3 genotypes V; Rio de Janeiro (2002) and Acre (2004): DENV-3 genotype III		Brazilian DENV-3 isolates grouped into two separate clades, comprising strains from Acre/Porto Velho/Rio de Janeiro (all genotype III) and Rondônia (genotype Very [South-East Asia/South Pacific]). Co-circulation of genotypes III and V was found in Rondônia

Source: first author, year [Ref]	Region/ geographical area	Study type	Study design	Data period: date range/ year	If study:				Summary of data presentation or results/conclusion
					No. patients/ population studied (M:F)	Diagnosis (DF/DHF/ DSS etc.)	Serotype/ genotype (lineage)	Age range	
			a Thermo Sequenase kit (USB Inc, Ohio, USA) on an ABI3100 device, with the Big-Dye7Terminator method(Applied Biosystems, Warrington, UK)						
Nogueira 2008 [44]	Brazil (São José do Rio Preto city)	Molecular characteris-ation and phylogenetic analysis	Comparative: blood samples from patients with DF and DHF symptoms were tested by RT-PCR; DENV-3 positive samples were sequenced (82 sequences) and compared with 52 reference sequences for phylogenetic reconstruction. The spatio-temporal dispersion pattern was analysed	2006	NS5 gene sequences from DENV-3 isolates from sera of patients diagnosed with DF/DHF (n=82), and from GenBank (n=52)	DF, DHF	DENV-3 (two lineages)		DENV-3 samples were closely related to strains circulating in Martinique and Brazil. Sixty samples formed a monophyletic group, representing lineage 1, and 22 samples formed lineage 2. The basic reproductive rate was 3.765 for lineage 1 and 3.093 for lineage 2. Both lineages appear to have split 1–3 years before the last collected sample, propagating in different regions of the city: north-western (lineage 1) and south-eastern (lineage 2)
Nogueira 2005 [37]	Brazil (Rio de Janeiro)	Description of laboratory and clinical findings of patients from the 2002 epidemic	Open: virus isolation and typing was performed, RNA extraction and RT-PCR was carried out, and dengue IgM-	January to June 2002	Acute- and convalescent-phase serum specimens, CSF, fresh tissues from patients (N=1,559)	DF, DHF	DENV-3	1–73 y (DENV-3 patients), 7–65 y (fatal cases only); even age distribution	831/1,559 (53.3%) confirmed dengue infection (fatal cases: 297); majority were DENV-3 serotype (99%); male:female: 1:1.08 (DENV-3 patients), 1:1.26 (fatal cases only). Neurological involvement in 1 patient with encephalitis,

Source: first author, year [Ref]	Region/ geographical area	Study type	Study design	Data period: date range/ year	If study:				Summary of data presentation or results/conclusion
					No. patients/ population studied (M:F)	Diagnosis (DF/DHF/ DSS etc.)	Serotype/ genotype (lineage)	Age range	
			capture was achieved by ELISA. Dengue infections were confirmed by virus isolation or viral RNA detection by RT-PCR, by IgM and/or IgG seroconversion, or by the demonstration of DENV antigen in formalised fixed autopsy tissues by immuno-histochemical tests					among all patients: 10.5% were 1–10 y; each 10-y age group thereafter accounted for 16.9–19.9% of all patients	confirmed by DENV-3 RNA in CSF
Nunes 2012 [49]	Brazil (northern: Boa Vista, Roraima; Manaus, Amazonas; Santarém, Pará; north-eastern: Salvador, Bahia)	Genetic characteris-ation and reconstruction of the spatio-temporal dispersion pattern of DENV-4 strains	Comparative Bayesian phylogeographic analysis on 98 full-length DENV-4 genomes compared with a similar analysis on 314 envelope gene sequences Sequencing method: nearly complete genome sequences were obtained by using high-throughput sequencing on a	2010–2011	Genomic or E-gene sequences from DENV-4 isolates (n=16) and GenBank (n=396)		DENV-4, genotype I DENV-4, genotype II		Two distinct DENV-4 genotypes (I, II) co-circulate in Brazil. Analysis confirmed the introduction of DENV-4 genotype I into Brazil from South-East Asia, and at least three introductions of DENV-4 genotype II in Brazil since 2002: two from Venezuela to Roraima, and one from Colombia to Amazonas. DENV-4 also appears to have been recently introduced into Pará State from the Caribbean region

Source: first author, year [Ref]	Region/ geographical area	Study type	Study design	Data period: date range/ year	If study:				Summary of data presentation or results/conclusion
					No. patients/ population studied (M:F)	Diagnosis (DF/DHF/ DSS etc.)	Serotype/ genotype (lineage)	Age range	
			GSFLX+ System(454LifeSciences,Branford,CT,USA). All 5′ and 3′ rapid amplification of cDNA ends amplicons were cloned into a plasmid bacterial system by using the TOPOTA Cloning Kit (Invitrogen) and bidirectionally sequenced using the ABIPrism BigDye Terminator v1.1 Cycle Sequencing Kit on an ABI Prism 3130 DNA analyser						

Source: first author, year [Ref]	Region/ geographical area	Study type	Study design	Data period: date range/ year	If study:				Summary of data presentation or results/conclusion
					No. patients/ population studied (M:F)	Diagnosis (DF/DHF/ DSS etc.)	Serotype/ genotype (lineage)	Age range	
Ocazionez 2006 [C]	Colombia (Bucaramanga and its metropolitan area, Santander state)	Longitudinal, serotype-specific study consisting of a surveillance study and a cross-sectional study	Surveillance study: patients coming to hospitals and private clinics were clinically examined and a blood samples was taken for routine laboratory tests. Cases were considered dengue laboratory-positive if an IgM-antibody-capture test was positive. Cross-sectional study: a standardised questionnaire was administered to collect demographic and clinical information. An acute sample was collected on the day of admission and a second sample 10–20 days later	Surveil-lance study: March 1998 to Decem-ber 2002; Cross-sectional study: May 2003 to Decem-ber 2004	Acute-phase serum samples from patients (N=1,452)	DF, DHF	DENV-1<br>DENV-2<br>DENV-3, genotype III<br>DENV-4		596/1,452 (41.0%) confirmed dengue infection; serotypes over the study period: DENV-3 subtype C (58.2%), DENV-2 (22.8%), DENV-1 (11.0%), DENV-4 (7.8%). At least 3 dengue serotypes have co-circulated in Bucaramanga and its metropolitan area since 1998. An annual increase in primary dengue infections (from 13.7 to 81.4%) correlated with frequency of DENV-3 (r=0.83; p=0.038). DENV-2 predominance in 2000–01 (17/35 isolates; 48.5%) coincided with highest DHF frequency (242/3192 cases; 7.6%). The 2001 outbreak was associated with re-introduction of DENV-3 (36%), which together with DENV-2 (40%) comprised the most prevalent serotypes, followed by DENV-4 (20%) and DENV-1 (4%). In 2002–03, DENV-3 became the most prevalent serotype (94.5%) and dengue activity remained high. DENV-3 predominance in 2003–04 (52/59 isolates; 88.1%) coincided with a decrease in the frequency of DHF cases (197/4423 cases; 4.4%), versus 2000–1. DHF was significantly more common in DENV-2- than DENV-3-

Source: first author, year [Ref]	Region/ geographical area	Study type	Study design	Data period: date range/ year	If study:				Summary of data presentation or results/conclusion
					No. patients/ population studied (M:F)	Diagnosis (DF/DHF/ DSS etc.)	Serotype/ genotype (lineage)	Age range	
									infected patients: 27.5 vs 10.9% (p<0.05)
Oliveira 2010 [34]	Brazil (Rio de Janeiro)	Phylogenetic analysis and sequencing	Comparative: DENV-2 isolates from these epidemic periods were subjected to sequencing and comparison (no further details provided)	1986–2008	DENV-2 isolates from the epidemic periods (number not given)	DF	DENV-2 American/Asian genotype (distinct lineages between isolates from the 1990/1998 epidemics and the 2007/2008 epidemics) Comparison of amino acid sequences from 1998 and 2008 strains found 6 amino acid substitutions in the envelope gene: V129I, L131Q, I170T,		DENV-2 isolates from 2007–8 formed a separate and distinct group from the 1990 and 1998 DENV-2 isolates, demonstrating a temporal circulation of genetically different viruses in Rio de Janeiro that could have arisen from local DENV-2 evolution since its introduction in 1990, or emergence of a new DENV-2 lineage

Source: first author, year [Ref]	Region/ geographical area	Study type	Study design	Data period: date range/ year	If study:				Summary of data presentation or results/conclusion
					No. patients/ population studied (M:F)	Diagnosis (DF/DHF/ DSS etc.)	Serotype/ genotype (lineage)	Age range	
							E203D, M340T and I380V		
PAHO 2015 [27]	WHO PAHO region	Surveillance data	Surveillance data	Ongoing surveil-lance data; data extracted for review period only	Country population	N/A	DENV-1,-2,-3 and -4	Country demo-graphics	Over the review period, the number of countries where more than one DENV serotype circulates steadily increased and all four serotypes were present and co-circulated in many countries
Peyrefitte 2003 [79]	Martinique (French West Indies)	Phylogenetic analysis and sequencing	Open: DENV infection identified by the presence of IgM, the elevation of specific IgG, or both, using DENV-specific ELISA.DENV-3 was identified by indirect immuno-fluorescence assay. Of 28 isolates, 5 were chosen randomly for partial sequencing (no data given)	1999–2002	November 1999 to December 2001: patients with dengue-like symptoms (n=97); September 2001 to January 2002: isolates from patients hospitalised with severe infection or outpatients (n=371)		DENV-3, subtype III		1999–2011: 97/97 (100%) confirmed dengue infection; serotype: DENV-3 (100%). 2001–2002: 134/371 (36.1%) confirmed dengue infection; serotypes: DENV-3 (99.3%), DENV-2 (0.7%). DENV-3 strains isolated in Martinique were closely related to each other, and with strains from Sri Lanka (isolated in 2000), the Philippines (reference strain D3PhilH87), Brazil, Guatemala and Mexico. This may indicate the existence of a Martinique-specific DENV-3 genotype, and a common origin in South-East Asia for all DENV-3 strains circulating in the American and Caribbean region
Peyrefitte 2005 [80]	Saint Martin island (French West Indies)	Phylogenetic analysis and sequencing	Comparative: DENV-3 was isolated (from blood samples) and partial genomic	October 2003 to April 2004	Patients with dengue-like symptoms (n=180)	Dengue-like syndrome	DENV-3 genotype III		108/180 (60%) confirmed dengue infection; 12 hospitalised; incidence rate: 180/29,000 (~0.62%); serotype (6 samples tested): DENV-3 (100%). Saint Martin

Source: first author, year [Ref]	Region/ geographical area	Study type	Study design	Data period: date range/ year	If study:				Summary of data presentation or results/conclusion
					No. patients/ population studied (M:F)	Diagnosis (DF/DHF/ DSS etc.)	Serotype/ genotype (lineage)	Age range	
			sequences were determined to evaluate the origin; spread and sequences were compared with those in the GenBank database Sequencing method: viral RNA was extracted; 2 overlapping viral cDNA fragments were generated; PCR products were directly sequenced with the BigDye Sequencing kit (Applera)						island DENV-3 isolates from 2003–4 were found to have a common origin with DENV-3 isolated in Martinique in 2001–2
Ramírez 2010 [64]	Venezuela	Phylogenetic analysis and sequencing	Open: 29 Venezuelan DENV-3 genotype III E-gene sequences representing strains isolated between 2000 and 2007 in seven different Venezuelan geographic locations, were aligned with 58 sequences from DENV-3 genotype III E-	2001–2008	E-gene sequences from DENV-3 genotype 3 isolates (n=29), and from GenBank (n=69)		DENV-3 genotype III (predominantly cluster A)<br><br>Amino acid substitution at position 329 of domain III of the E protein (alanine to valine) in almost all E proteins from Cluster A strains		DENV-3 genotype III strains belonging to 3 different clusters (A to C) were observed in Venezuela, revealing several introduction events. The evolutionary rate for cluster A strains circulating in Venezuela (8.48 x 10^{-4} substitutions/site/year) is similar to that previously established for this genotype in other regions of the world, suggesting a lack of correlation among DENV-3 genotype III substitution rate and ecological pattern of virus spread

Source: first author, year [Ref]	Region/ geographical area	Study type	Study design	Data period: date range/ year	If study:				Summary of data presentation or results/conclusion
					No. patients/ population studied (M:F)	Diagnosis (DF/DHF/ DSS etc.)	Serotype/ genotype (lineage)	Age range	
			gene of DENV isolated in Latin America and 11 DENV-3 sequences from strains isolated elsewhere representing other DENV-3 genotypes Sequencing method: viral RNA was extracted and reverse transcribed to cDNA; amplicons were purified and the sequence reaction was carried out using the Big Dye DNA sequencing kit (PerkinElmer)						
Regato 2008 [63]	Ecuador	Phylogenetic analysis and sequencing	Comparative: sequencing and phylogenetic analysis of 23 Ecuadorian DENV NS5 sequences plus 56 comparable sequences from DENV strains isolated elsewhere Sequencing method: DENV-	2000–2007	NS5 sequences from DENV isolates from sera of patients (n=23), and from GenBank (n=56)	Dengue-like syndrome	DENV-1 DENV-2, American genotype DENV-3 DENV-4		23/23 (100%) confirmed dengue infection. Ecuadorian strains were found to be closely related to DENV isolates of Caribbean origin. Although the Ecuadorian strains do not cluster with the Brazilian DENV-3 strain (EF110568) included in this study, revealing a different evolutionary history, very recent studies suggest that DENV-3 might have also

Source: first author, year [Ref]	Region/ geographical area	Study type	Study design	Data period: date range/ year	If study:				Summary of data presentation or results/conclusion
					No. patients/ population studied (M:F)	Diagnosis (DF/DHF/ DSS etc.)	Serotype/ genotype (lineage)	Age range	
			positive serum samples underwent RT-PCR. The sequence reaction was carried out using the Big Dye DNA sequencing kit (Perkin-Elmer) on a 373 DNA sequencer apparatus (Perkin-Elmer)						been introduced to Brazil from the Caribbean region
Roca 2009 [57]	Bolivia	Phylogenetic analysis and sequencing	Comparative: isolates were sequences and compared with 2 E-gene sequences available in GenBank and previously produced from Bolivian strains isolated in 1997 Sequencing method: the complete viral E-gene was then amplified using PCR; amplicons were directly sequenced and viral nucleotide sequences of each dengue serotype were aligned using	1998–2008	E-gene sequences from DENV isolates (n=64; DENV-1: 16, DENV-2: 23, DENV-3: 25), and from GenBank (n=2)		DENV-1, American–African genotype V DENV-2, Asian-American genotype DENV-3, genotype III		In Bolivia, closely related DENV viruses circulated during the several consecutive years of the study (5, 6 and 6 years for DENV-1, DENV-2, and DENV-3, respectively). Co-circulation of up to 3 serotypes was observed. Emergence of new variants, distinct from those identified during previous outbreaks, occurred for DENV-1 (2007 outbreak) and DENV-2 (2001 outbreak). In all cases, DENV viruses likely originated from neighbouring countries

Source: first author, year [Ref]	Region/ geographical area	Study type	Study design	Data period: date range/ year	If study:				Summary of data presentation or results/conclusion
					No. patients/ population studied (M:F)	Diagnosis (DF/DHF/ DSS etc.)	Serotype/ genotype (lineage)	Age range	
			ClustalX together with relevant sequences retrieved from GenBank						
Rodriguez-Roche 2005 [78]	Cuba (Havana)	Phylogenetic analysis and sequencing	Open: the complete E-gene sequences of isolates from both outbreaks were determined. To assist in this analysis, a DENV-3 strain representing the 1994 Nicaraguan epidemic was sequenced Sequencing method: viral RNA was extracted and the E-gene was amplified using RT-PCR. Double-stranded sequencing of the E-gene was conducted on an ABI sequencer	2000–2001	DENV-3 E-gene sequences from patient sera (n=2) and the spleen of a deceased patient (n=1), and a reference DENV-3 strain representing the 1994 Nicaraguan epidemic (n=1)		DENV-3, genotype III		An Asian DENV-3 virus assigned to genotype III appears to have evolved *in situ* and been circulating in the Caribbean region since 1994. By comparing the amino acid sequences of the Cuban isolates with other DENV-3 strains assigned to genotype III, several distinct amino acid replacements were noted
Rodriguez-Roche 2012 [58]	Venezuela (Maracay, Aragua state)	Phylogenetic analysis and sequencing	Open: 21 DENV full-length genomes representing all 4 serotypes were amplified and sequenced	Novem-ber 2006 to April 2007	Genomic sequences (n=21) from DENV isolates (n=31) from acute-phase	Severe and non-severe DF	DENV-1, genotype III (three clusters) DENV-2, American genotype (1987 isolate)		31/50 (62%) confirmed dengue infection; serotypes: DENV-1 (n=10), DENV-2 (n=10), DENV-3 (n=2), DENV-4 (n=9). Only DENV-2 was associated with severe disease, and only one

Source: first author, year [Ref]	Region/ geographical area	Study type	Study design	Data period: date range/ year	If study:				Summary of data presentation or results/conclusion
					No. patients/ population studied (M:F)	Diagnosis (DF/DHF/ DSS etc.)	Serotype/ genotype (lineage)	Age range	
			directly from the serum samples<br>Sequencing method: viral RNA was extracted and, following PCR, direct sequencing of PCR products was carried out using an Applied Biosystems BigDye ddNTP capillary sequencer. The chromatograms from capillary sequencing were assembled into a specimen consensus sequence using SeqScape version 2.5 (Applied Biosystems)		patient sera (n=50)		DENV-2, American-Asian genotype (cluster A, isolates from 1990s; cluster B, isolates from 2006–2007)<br>DENV-3, genotype III (all but two isolates clustered together)<br>DENV-4, genotype II (distinct co-circulating lineages)		genotype appeared to be circulating for each DENV serotype. However, extensive viral genetic diversity was found in DENV isolated from the same area during the same period, indicating significant *in situ* evolution since the introduction of these genotypes. Evidence suggests that multiple introductions of DENV have occurred from the Latin American region into Venezuela and vice versa
Romano 2010 [35]	Brazil (São Paulo state: Guarujá, Santos, Saõ Vicente)	Phylogenetic analysis and sequencing	Open: sequencing and phylogenetic analysis of partial E viral genes<br>Sequencing method: viral RNA was isolated and a 665 bp of E-region	2010	Patients with clinically suspected DF (n=18)<br><br>(6:12)	DF, DSS, DHF	DENV-2, American/Asian genotype	1 wk–86 y.	17/18 (94.4%) confirmed dengue infection (DHF: 1, DSS: 1);<br>Isolated strains were confirmed as DENV-2 American/Asian genotype, closely related to the strain that circulated in Rio de Janeiro during the 2007–08 epidemic, but distinct from those of earlier DENV-2 epidemics in Brazil. The most

Source: first author, year [Ref]	Region/ geographical area	Study type	Study design	Data period: date range/ year	If study:				Summary of data presentation or results/conclusion
					No. patients/ population studied (M:F)	Diagnosis (DF/DHF/ DSS etc.)	Serotype/ genotype (lineage)	Age range	
			corresponding to nucleotides 1,857 to 2,522 of DENV-2 complete genome was amplified by PCR. Sequencing reactions were performed with BigDye Terminator kit						recent common ancestor of the American/Asian genotype and the São Paulo and Rio de Janeiro monophyletic cluster is estimated to have appeared around 1970 and 2000, respectively
Santiago 2012 [81]	Puerto Rico	Phylogenetic analysis and sequencing	Open: a multiple sequence alignment of 124 complete coding sequences was made. Aligned sequences included the 92 Puerto Rico isolates from this study, an additional 22 South/Central American strains sequenced as part of the Broad Institute's Genome Resources in Dengue Consortium, and an additional 10 international strains obtained from GenBank Sequencing	1998–2007	Genomic sequences from DENV-3 isolates from human sera (n=92), and from GenBank/ Broad Institute's Genome Resources in Dengue Consortium (n=32)		DENV-2<br>DENV-3		Two primary DENV-3 lineages (clades 1 and 2) were identified in Puerto Rico. Clade 1 consisted of two subclades (1A, 1B), both closely related to international DENV-3 isolates. Clade 2 consisted of multiple subclades, all of which emerged rapidly and almost simultaneously from the parent population. Several subclades (2A, 2E, 2F) also exhibited rapid, sustained diversification. The high mutation rates and rapid replication of DENV-3 may have produced a highly heterogeneous population structure at each lineage

Source: first author, year [Ref]	Region/ geographical area	Study type	Study design	Data period: date range/ year	If study:				Summary of data presentation or results/conclusion
					No. patients/ population studied (M:F)	Diagnosis (DF/DHF/ DSS etc.)	Serotype/ genotype (lineage)	Age range	
			method: bidirectional Sanger sequencing was performed on pooled cDNA or PCR amplicons using an ABI 3730 automated sequencer. The resulting sequence was trimmed at the ends to remove both low-quality and primer sequence and assembled resulting reads using Broad Institute's AV454 assembly algorithm. Consensus assemblies were annotated by the Broad Institute using an in-house annotation algorithm						
Sharp 2013 [75]	Puerto Rico	A retrospective analysis of suspected dengue cases reported to surveillance systems	Virus confirmed by RT-PCR and indirect immuno-fluorescence. Viral RNA extracted from culture supernatants	1 January and 31 December 2010	26,766 suspected dengue cases (53:47)	Of 12,048 laboratory -positive cases, sufficient clinical data were provided	7,426 RT-PCR-positive specimens: DENV-1 (69.0%); DENV-2 (7.3%); DENV-3 (0.1%);	Median age range (suspected cases): 18y (5 days–102 years)	49.7% had dengue with warning signs, 11.1% had severe dengue, and 40 died. Approximately 21% of cases were primary DENV infections; 1–4 year olds were the only age group for which primary infection was

Source: first author, year [Ref]	Region/ geographical area	Study type	Study design	Data period: date range/ year	If study:				Summary of data presentation or results/conclusion
					No. patients/ population studied (M:F)	Diagnosis (DF/DHF/ DSS etc.)	Serotype/ genotype (lineage)	Age range	
			using the M48 BioRobot System (Qiagen; Valencia, CA). The E glycoprotein gene was amplified and sequenced. Multiple sequence alignment performed in MEGA 5 (megasoftware.net). Phylogenetic trees rendered using the maximum likelihood method			to classify 74.0% as DF and 2.4% as DHF	DENV-4 (23.6%)		more common than secondary. Individuals infected with DENV-1 were 4.2 (95% confidence interval [CI]: 1.7–9.8) and 4.0 (95% CI: 2.4–6.5) times more likely to have primary infection than those infected with DENV-2 or -4, respectively. Sequencing and phylogenetic analyses of randomly selected DENV isolates showed that DENV-1 belonged to the American-African genotype (genotype V) to a clade distinct from virus isolated during the 1998 Puerto Rico epidemic. Close ascendants of the 2010 DENV-1 clade had been circulating in Puerto Rico and the Caribbean since at least 2006. DENV-2 sequencing: the virus belongs to clade 1B of the American-Asian genotype (genotype IIIb; DENV-4 belonged to the Indonesian genotype (genotype II), but was distinct from virus isolated in 1998. Viruses closely-related to the 2010 DENV-4 isolate were first detected in Puerto Rico in 2004 [70]
Usme-Ciro 2008 [60]	Colombia (Antioquia, Caquetá, Guaviare, Huila, La Guajira, Meta, Norte de Santander,	Phylogenetic analysis and sequencing	Open: serotype confirmed using serotype-specific monoclonal antibodies	2002–2005	E-gene sequences from DENV-3 isolates (n=32)		DENV-3, genotype I DENV-3, genotype III		DENV-3 appeared to be circulating in Colombia since 2002. Importantly, genotype I (South-East Asia/South Pacific genotype) was

Source: first author, year [Ref]	Region/ geographical area	Study type	Study design	Data period: date range/ year	If study:				Summary of data presentation or results/conclusion
					No. patients/ population studied (M:F)	Diagnosis (DF/DHF/ DSS etc.)	Serotype/ genotype (lineage)	Age range	
	Putumayo, San Andrés, Santander)		Sequencing/ phylogenetic analysis Sequencing method: viral RNA was extracted and subjected to RT-PCR. PCR p were purified and sequencing reactions on both strands were performed with the ABI Prism Dye Terminator Cycle Sequencing Ready Reaction Kit (Applied Biosystems, USA)						detected for the first time in the Americas, co-circulating with genotype III (Indian genotype) in three states (Guaviare, Huila, La Guajira). Co-circulation of different genotypes undergoing intra-serotype antigenic variation, and the resulting differential serological and immune responses, may be a factor in the association between DENV-3 infection and disease severity, and account for the high epidemiological impact of DENV-3 in the Americas
Uzcategui 2003 [65]	Venezuela (Aragua state)	Molecular epidemio-logical	Comparative: determination of the complete sequence of the E-gene of 15 Venezuelan DENV-3 viruses isolated during 2000 and 2001 Sequencing method: viral RNA was extracted. Nucleotides from position 716 in the prM region to 2013 in the NS1	2000–2001	E-gene sequences from isolates from sera of patients with DF (n=11) and suspected DHF/DSS (n=4), strains from the 1995 Mexican DENV-3 (n=1) and 1960s Puerto Rican (n=2) outbreaks, and	DF, DHF	DENV-3, genotype III		The DENV-3 strain circulating in Venezuela appears to be closely related to isolates that were previously present in Panama and Nicaragua in 1994, which have since spread throughout Central American countries and Mexico. It was most closely related to the Mexican isolate from the 1995 epidemic (100% bootstrap support) and a Brazilian isolate from 2000.This study confirms previous reports that the DENV-3 strain currently circulating in the Americas is

Source: first author, year [Ref]	Region/ geographical area	Study type	Study design	Data period: date range/ year	If study:				Summary of data presentation or results/conclusion
					No. patients/ population studied (M:F)	Diagnosis (DF/DHF/ DSS etc.)	Serotype/ genotype (lineage)	Age range	
			region of the DENV-3 genome encoding the prM/M, E and NS1 genes were amplified using PCR. Double-stranded sequencing of the prM/M and E-gene was performed on an ABI sequencer		GenBank (n=50)				related to the strain that caused DHF epidemics in Sri Lanka and India in 1989–91 (genotype III)
Villabona-Arenas 2009 [61]	Colombia (states of Norte de Santander, Santander, Valle del Cauca)	Phylogenetic analysis and sequencing	Comparative: viruses were isolated in and sub-typed both by indirect immunofluor-ecescence with monoclonal antibody (CDC, Puerto Rico) or RT-PCR Sequencing method: viral RNA was extracted and cDNA was amplified by PCR. PCR products were purified and sequenced under BigDye Terminator cycling conditions by using automatic	2001–2007	E-gene sequences from DENV-3 strains (n=21) and GenBank (n=98; from 13 Latin American countries and Sri Lanka)		DENV-3, genotype III (clades III and IV)		This study confirms previous reports showing that Colombian isolates are closely related to DENV-3 genotype III. Colombian DENV-3 strains seem to have been introduced from Ecuador, Peru and Venezuela, but not from Argentina, Brazil, Paraguay or Central American countries. Colombian isolates clustered apart from Brazilian DENV-3 isolates, which were associated with a significant number of DHF cases and fatalities, suggesting that DENV-3 genotype III strains circulating in Latin America may exhibit different pathogenic potential

Source: first author, year [Ref]	Region/ geographical area	Study type	Study design	Data period: date range/ year	If study:				Summary of data presentation or results/conclusion
					No. patients/ population studied (M:F)	Diagnosis (DF/DHF/ DSS etc.)	Serotype/ genotype (lineage)	Age range	
			sequencer 3730 · l in a commercial manufacturer (Macrogen, Geumchungu, Seoul, Korea) Sequence assembly was performed with the Lasergene package v7.0 (DNASTAR)						

Abbreviations: C, capsid; CSF, cerebrospinal fluid; DENV, dengue virus; DF, dengue fever; DHF, dengue haemorrhagic fever; DSS, dengue shock syndrome; E, envelope; ELISA, enzyme-linked immunosorbent assay; GTR, general time-reversible; IgG, immunoglobulin G; IgM, immunoglobulin M; M, membrane; N/A, not available; NS, non-structural; ORF, open reading frame; PCR, polymerase chain reaction; prM, pre-membrane; qRT-PCR, quantitative reverse transcriptase-polymerase chain reaction; RT-PCR, reverse transcriptase-polymerase chain reaction; SE, standard error; UTR, untranslated region.

Sources included in the review and cited in Supplementary Table S1 but not cited in the paper

A de Araujo JM, Nogueira RM, Schatzmayr HG, Zanotto PM, Bello G. Phylogeography and evolutionary history of dengue virus type 3. Infect Genet Evol 2009;9: 716-725.

B Huhtamo E, Comach G, Sierra G, Camacho DE, Sironen T, Vapalahti O, et al. Diversity and composition of dengue virus type 2 in Venezuela. Epidemiol Infect 2013;141: 1816-1822.

C Ocazionez RE, Cortes FM, Villar LA, Gomez SY. Temporal distribution of dengue virus serotypes in Colombian endemic area and dengue incidence. Re-introduction of dengue-3 associated to mild febrile illness and primary infection. Mem Inst Oswaldo Cruz 2006;101: 725-731.
